# The shadow of the past: Convergence of young and old South American desert lizards as measured by head shape traits

**DOI:** 10.1002/ece3.4548

**Published:** 2018-11-26

**Authors:** César Aguilar‐Puntriano, Luciano J. Avila, Ignacio De la Riva, Leigh Johnson, Mariana Morando, Jaime Troncoso‐Palacios, Perry L. Wood, Jack W. Sites

**Affiliations:** ^1^ Departamento de Herpetología Museo de Historia Natural de San Marcos (MUSM) Lima Perú; ^2^ Instituto Antonio Raimondi, Facultad de Ciencias Biológicas Universidad Nacional Mayor de San Marcos (UNMSM) Lima Perú; ^3^ Instituto Patagónico para el Estudio de los Ecosistemas Continentales (IPEEC‐CONICET) Puerto Madryn, Chubut Argentina; ^4^ Museo Nacional de Ciencias Naturales Madrid Spain; ^5^ Department of Biology, M. L. Bean Life Science Museum Brigham Young University (BYU) Provo Utah; ^6^ Programa de Fisiología y Biofísica, Instituto de Ciencias Biomédicas (ICBM), Facultad de Medicina Universidad de Chile Santiago Chile; ^7^ Biodiversity Institute, Department of Ecology and Evolutionary Biology The University of Kansas Lawrence Kansas

**Keywords:** *Ctenoblepharys adspersa*, *Liolaemus*, repeated evolution, South America

## Abstract

Convergence is a pervasive phenomenon in the Tree of Life, and evolution of similar phenotypes sharing the same environmental conditions is expected in phylogenetically closely related species. In contrast, contingent factors are probably more influential in shaping phenotypic diversity for distantly related taxa. Here, we test putative convergent evolution of lizard head morphologies among relatively closely related desert dwelling *Liolaemus* species, and the very distantly related *Ctenoblepharys adspersa*. We estimated a multilocus time‐calibrated phylogeny of 57 species of South American liolaemus lizards, based on seven molecular markers. We collected head shape data for 468 specimens, and used three phylogenetic comparative methods (SURFACE, CONVEVOL, and WHEATSHEAF index) to test for and estimate the strength of convergence. We found strong evidence for convergence among Pacific desert lizard *C. adspersa*,* Liolaemus audivetulatus*,* Liolaemus insolitus*,* Liolaemus poconchilensis*,* Liolaemus stolzmanni*, and a candidate species (*Liolaemus* “Moquegua”). Our results suggest that, despite the long divergence and phylogenetic distance of *C. adspersa* with respect to convergent *Liolaemus* species, natural selection was probably more important than historical contingency in shaping phenotypic evolution in these desert lizards.

## 1 INTRODUCTION

Evolutionary convergence is a pervasive phenomenon in the Tree of Life and can be defined as the repeated, independent evolution of the same trait (or complex of traits) in two or more clades (McGhee, 2011). Two possible goals in the study of evolutionary convergence are its identification (whether convergence is present) and quantification (estimating its frequency and strength; Arbuckle & Speed, 2017). The frequency of convergence is quantified by enumerating the cases in a group of taxa, while the strength of convergence estimates how similar is/are the trait(s) of the convergent taxa while taking phylogenetic distance into account.

Another important issue in studying convergence is whether natural selection and constraints can erase the contingent nature of evolution. Natural selection and constraints should be more prominent in shaping similar adaptive phenotypes of related species (e.g. species of the same genus), but historical contingencies may lead to a more significant imprint when taxa are not closely related (Ord & Summers, 2015). However, because the number of possible forms is finite, even phylogenetically distant taxa will evolve the same adaptations when exposed to the same selective pressures. So, under similar environmental conditions, closely related as well as distantly related taxa may either (a) evolve similar phenotypes independently of past events; or (b) not show repeated evolution because of historical contingencies (Ord & Summers, 2015).

**Figure 1:**
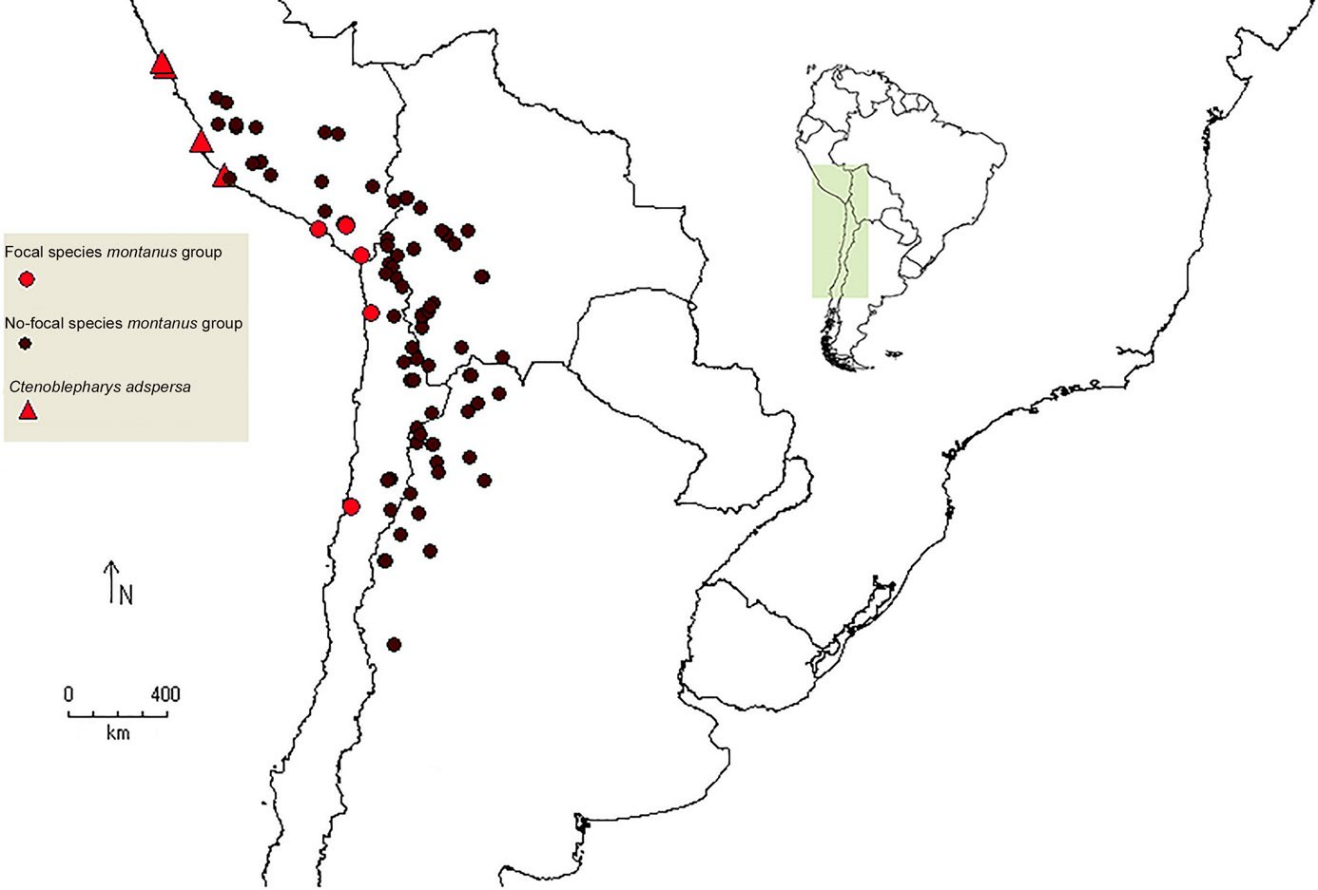
Distribution map of *Liolaemus* species of the *Liolaemus montanus* group. Focal species (*Liolaemus audituvelatus*,* Liolaemus insolitus*,* Liolaemus poconchilensis*,* Liolaemus stolzmanni*,* Liolaemus* “Moquegua” and *Ctenoblepharys adpersa*) are represented by red symbols, and non‐focal species by black dots

**Figure 2:**
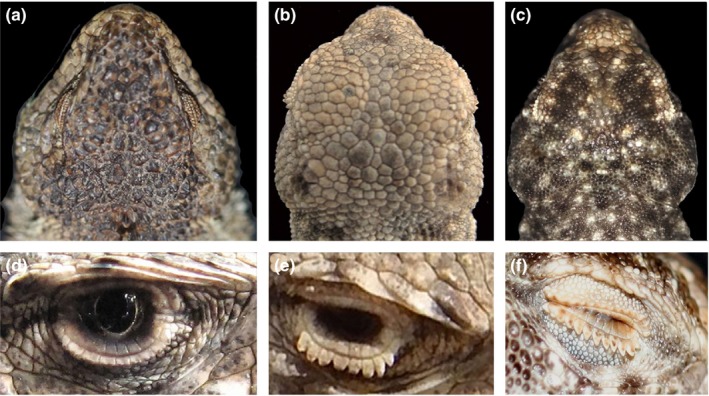
Morphological traits in non‐focal (left) versus focal (center and right) species: (a) (*Liolaemus melanogaster*), (b) (*Liolaemus poconchilensis*) and (c) (*Ctenoblephrys adspersa*) show differences in head shapes; (d,e,f) show eyes framed by reduced ciliary scales in C (*Liolaemus* “Nazca”) versus conspicuous comb‐like ciliaries in (d) (*Liolaemus poconchilensis*) and (f) (*C. adspersa*)

Here, we test for convergence in South American desert lizards of the *Liolaemus montanus* group (Figure 1). Some species in this group have toad‐like (“phrynosauroid”) head shapes and pronounced serrated combs formed by the projecting outer ciliary scales, which are lacking in other closely related members of this species group (Figure 2). Other species and populations have a similar toad‐like head, but lack the pronounced serrated combs. These “phrynosauroid” lizards and similar forms inhabit the extremely arid desert environments (mean annual rainfall ~1–15 mm) of the South American Pacific coast (Rundel, Villagra, Dillon, Roig‐Juñent, & Debandi, 2007), in contrast to the remaining, mostly Andean, species of the *L. montanus* group. Moreover, these species resemble distantly related taxa present in the same arid deserts, the monotypic *Ctenoblepharys adspersa* (Tschudi, 1845) (Liolaemidae).

The taxonomic history of these desert species is a good example of how putative convergence has confused taxonomists. “Phrynosauroid” lizards from the *L. montanus* group (*Liolaemus audituvelatus* [Núñez & Yáñez, 1983], *Liolaemus erroneous* [Núñez & Yáñez, 1983], *Liolaemus poconchilensis* Valladares, 2004, *Liolaemus stolzmanni* [Steindachner 1891],* Liolaemus torresi* [Núñez, Navarro, Garín, Pincheira‐Donoso & Meriggio, 2003]) are different from each other, but they were formerly included in the genus *Phrynosaura* and *Ctenoblepharys* (Donoso‐Barros, 1971, 1972 ). In contrast, *Liolaemus insolitus* Cei & Pefaur, 1982 was also considered so distinct from *Liolaemus* but included in another genus (*Abas*; Núnez & Yánez, 1984). In more recent studies, these “new” genera were later rejected, and all species except *C. adspersa* were returned to *Liolaemus* (Etheridge, 1995; Lobo, Espinoza, & Quinteros, 2010). Moreover, there is not a comprehensive phylogeny of the *L. montanus* group which includes “Phrynosauroid” lizards and related forms, thus preventing a formal test of convergence and its strength.

The arid conditions and sandy substrates of the Peruvian and Atacama deserts likely have exerted strong selective pressures for the evolution of convergent phenotypes among these taxa. Although toad‐like head shapes with enlarged ciliaries in “Phrynosauroid” lizards have been mentioned together as convergent traits for living in desert conditions (Valladares, 2004), they may reflect different aspects of their natural histories. Toad‐like head shapes may be related to a diet composed largely of small preys, as documented for North American *Phrynosoma* lizards (Meyers, Herrel, & Nishikawa, 2006; Meyers, Nishikawa, & Herrel, 2018), in comparison with other desert living and hard‐preyed specialist lizards of the genera *Gambelia* and *Crotaphytus* (Modlin, 2018). However, dietary data for *C. adspersa* and putatively convergent *Liolaemus* are limited. A high percentage of Hymenoptera and small Coleoptera was found in *C. adspersa* and *L. insolitus* stomach contents, respectively (Cei & Pefaur, 1982; Pérez & Balta, 2007). Given that small preys comprise most of the diets in *C. adspersa* and the putatively convergent *Liolaemus*, the repeated evolution of their distinct head morphology may reflect a loss of morphological traits related to the acquisition and processing of a more generalist diet.

In contrast, strongly enlarged ciliaries may protect the eyes from sand (Etheridge, 2000) while burrowing, or inside a burrow. Burrowing in loose sand is known in this convergent group in *C. adspersa* (CA, personal observation), and phrynosauroid *Liolaemus* may use the projecting ciliaries to deflect sand from their eyes when they use burrows (J. Troncoso‐Palacios, personal observation; Díaz‐Vega, 2015).

The aims of this paper are to: (a) test the monophyly of “phrynosauroid” lizards and related forms of the *L. montanus* group and estimate their divergence times using a fossil‐calibrated multilocus dataset; and (b) test for phenotypic convergence in Southern Pacific desert lizards using quantitative head traits. If the phylogenetic signal of natural selection overrides historical contingency, we should expect *C. adspersa* and “phrynosauroid” forms of the *L. montanus* group be very similar in head traits. Alternatively, if stochastic events dominated the evolutionary history of this clade, we should expect “phrynosauroid” lizards and related forms of the *L. montanus* group to be more similar to each other than to *C. adspersa*.

## 2 MATERIAL AND METHODS

### 2.1 Phylogenetic analyses

Details of field sampling, laboratory procedures, specimen assignment and locality data are summarized in Supporting Information Appendix S1. Sequences were aligned in the MUSCLE (Edgar, 2004) plugin in GENEIOUS®PRO v5.6.6 (Kearse et al., 2012), and protein coding sequences were translated to check for premature stop codons. Bayesian information criteria in JMODELTEST v2.1.3 (Darriba, Taboada, Doallo, & Posada, 2012) was used to identify the best‐fit models of molecular evolution. Maximum likelihood (ML) analyses were performed using RAXML v.8 (Stamatakis, 2014) partitioned by gene, and 1,000 standard bootstrap replications were estimated using the rapid hill‐climbing algorithm (Stamatakis, Hoover, & Rougemont, 2008) in the Cyber Infrastructure for Phylogenetic Research (CIPRES; Miller, Pfeiffer, & Schwartz, 2010). The ML tree is shown in Supporting Information Figure S1.

To estimate divergence times, a concatenated Bayesian tree (BT) was generated and calibrated as in Aguilar, Wood, Belk, Duff, and Sites (2017). This analysis was implemented in BEAST v2.4.6 (Bouckaert et al., 2014) using the bModelTest (Bouckaert & Drummond, 2017) to simultaneously generate and explore the model substitutions across space to estimate model parameters, and to generate a fossil‐calibrated phylogeny. We implemented 10 independent runs for 100 million generations, sampling every ten thousand generations, and we checked for convergence of the runs using TRACER v1.6 (Drummond, Suchard, Xie, & Rambaut, 2012) to ensure effective samples sizes (ESS) were >200. A Yule speciation prior and a log‐normal relaxed clock model were applied. We discarded 10% of the trees and log files as burn‐in, and the remaining trees were combined using LOGCOMBINER v1.8.0 and sampled at a lower frequency, resulting in 10,000 trees. A maximum clade credibility tree (MCC) was then constructed using TREEANNOTATOR v1.8 (Drummond et al., 2012), and keeping mean and 95% confident intervals for node ages.

### 2.2 Morphological data and analyses

Shape analyses were performed using principal component (PC) analysis after a Generalized Procrustes approach. Procustes and PCA analyses were performed using MORPHOJ v1.03d (Klingenberg, 2011), and PC scores were extracted using the GEOMORPH package (Adams & Otarola‐Castillo, 2013) in R (R Development Core Team, 2014). The first three principal components explained 74.8% of the variance and were retained for further analyses (Supporting Information Appendix S3). We also estimated average of PC1–PC3 scores for specimens representing each species (Supporting Information Appendix S3). The use of PC scores in phylogenetic comparative studies has been criticized when only a few PC axes have been selected (Adams & Collyer, 2017; Uyeda, Caetano, & Pennell, 2015), but it is the best available approach for our purposes. Geometric morphometric data are necessarily reflecting one or more multivariate traits (Collyer, Sekora, & Adams, 2015); these are usually reduced using PC analysis, and the first PC axes that explain most of the variance are employed instead of the original data (e.g. Muschick, Indermaur, & Salzburger, 2012; Esquerre & Keogh, 2016).

To examine convergence traits in the *L. montanus* group, the BT was combined with head shape data. Ten landmarks on the dorsal head view (Aguilar et al., 2017; Supporting Information Appendix S3) of 468 lizards representing 57 species (Supporting Information Appendix S3) were set on digital pictures using TPSdig v1.4 (Rohlf, 2004). Landmarks (number in parentheses) were set on the tip of the snout (1), nostrils in each side (2, 3), beginning of the first superciliary scale in each side (4, 6), end of the last superciliary scale in each side (5, 7), interparietal scale (8), dorsal widest part of the head at the level of anterior margin of the external ear in each side (9, 10). These landmarks were selected to represent shape differences in different, but homologous areas of the head. Additional homologous landmarks were difficult to set because they were not repeatable across the very distantly related taxa (e.g. *C. adspersa* and other *Liolaemus* species).

We tested for deviation of head symmetry using Procrustes ANOVA in MORPHOJ v1.03d (see Supporting Information Appendix S3 for details). For each species, only adults of each sex (as assessed by larger snout vent length) were used to avoid ontogenetic allometric bias. When sample sizes within species allowed comparisons between sexes, we tested for sexual dimorphism for each species using the PCA scores of the first two PCs. Because males and females did not cluster separately in each species tested, data of both sexes were pooled together for further analyses. We also performed a discriminant function analysis using Procrustes coordinates to test for shape differences between sexes and all species, but we did not find strong significant differences between males and females (Supporting Information Appendix S3). Because specimens of *Liolaemus lentus* and *Phymaturus sitesi* were not available, we used another species of the same group (*Liolaemus pseudoanomalus*) or same genus (*Phymaturus patagonicus*), respectively.

### 2.3 Convergence analyses

The BT was pruned using R package APE v5.1 (Paradis, Claude, & Strimmer, 2004) to include only taxa for which morphological data were available (see above). Evolutionary changes in head shape were reconstructed by using the PC scores onto the BT phylogeny, and then employing a squared‐change parsimony analysis (Maddison, 1991) method in MORPHOJ. This method was selected to visualize the evolutionary changes between the reconstructed head shape of the focal taxa and their most recent common ancestor by deformation grids and displacements of landmarks.

Three convergence analyses were performed using PC1‐PC3 axes. The first analysis was performed using the R package SURFACE (Ingram & Mahler, 2013); this algorithm employs an Ornstein‐Uhlenbeck (OU) process to identify cases of convergence without the a priori designation of convergent taxa. The method has a forward phase in which selective regimes are inferred using a phylogenetic tree and quantitative traits, and a reverse phase in which taxa having the same (convergent) regime are identified. In the forward phase, selective regimes are added to a Hansen model (Hansen, 1997) and then further regime shifts (models) are added in a stepwise process. Model performance is evaluated using corrected Akaike information criterion (AICc). In the reverse phase, all selective regimes obtained in the first phase are combined in a pairwise manner and collapsed into a shared regime. This procedure is repeated until no more stepwise combinations improve the models, and convergent (collapsed) regimes are estimated (again using AIC). The SURFACE model with convergent regimes (OUc) was compared with a model with multiple non‐convergent regimes (OUnc), and simpler stochastic models such a single regime (OU1) and Brownian motion (BM) models.

Focal taxa (*C. adspersa*,* L. audituvelatus*,* L. insolitus*,* L. stolzmanni*,* L. poconchilensis* and *Liolaemus* “Moquegua”), and two subsets (one excluding *L. *“Moquegua,” and another excluding *L. insolitus* and *L. *“Moquegua”) were used to estimate convergent metrics with CONVEVOL (Stayton, 2015a). This method estimates four distances (C1, C2, C3, C4) and one frequency‐based (C5) measure of convergence. C1 is based on the idea that the more dissimilar the ancestors, and the more similar the descendants, the stronger is the convergence. C1 represents the proportion of the maximum distance between two lineages that has been brought together by subsequent evolution, and ranges from 0 to 1 as convergence increases. A value of 1 indicates that lineages are fully convergent, and a value of 0 means that lineages are phenotypically different, and convergence is absent. C2 is another measure representing the absolute amount of evolution that has occurred during convergence, with larger values indicating greater convergence. C3 and C4 are based on C2 and allow comparison between datasets (in contrast to within datasets). C3 is the proportion between C2 and the total amount of evolutionary change along the lineages leading from the common ancestor of the convergent taxa to those taxa. C4 is the proportion between C2 and the total amount of evolution in the entire clade defined by the common ancestor of the convergent taxa (Stayton, 2015a).

C5 is a frequency‐based measure and is defined as the number of focal taxa that reside within a limited but convergent region of a phylomorpho‐space (the phylogenetic connections between taxa represented graphically in a plot of morphological space).

Statistical tests of convergence as measured by C1, C2, C3, and C4 were evaluated using 500 evolutionary simulations via a BM model. Specifically, we tested whether the simulated measures are significantly different from the observed values. In the same way, the statistical significance of convergence as measured by C5 was tested using 500 simulations. Results of all tests were considered significant at a *p*‐value ≤0.05.

We implemented a WHEATSHEAF analysis to measure the strength of convergent evolution in focal taxa and subsets (see above), as implemented in the R package WINDEX (Arbuckle & Minter, 2015; Arbuckle, Bennett, & Speed, 2014). This index calculates the similarity of focal (convergent) species to each other and the separation in phenotypic space of the focal group from non‐convergent species, all corrected for phylogenetic relatedness. Convergence is stronger when focal species are more phenotypically similar to each other but phylogenetically more distant, relative to the non‐focal species. Convergent focal taxa in SURFACE and CONVEVOL were used to estimate the WHEATSHEAF Index and 95% confidence intervals. The null hypothesis that the observed WHEATSHEAF index is no higher than expected by chance is rejected when *p* ≤ 0.05 (indicating exceptionally strong convergence). Expected WHEATSHEAF indexes were derived from 1,000 bootstrap replications.

## 3 RESULTS

### 3.1 Phylogenetic relationships and divergence times

**Figure 3:**
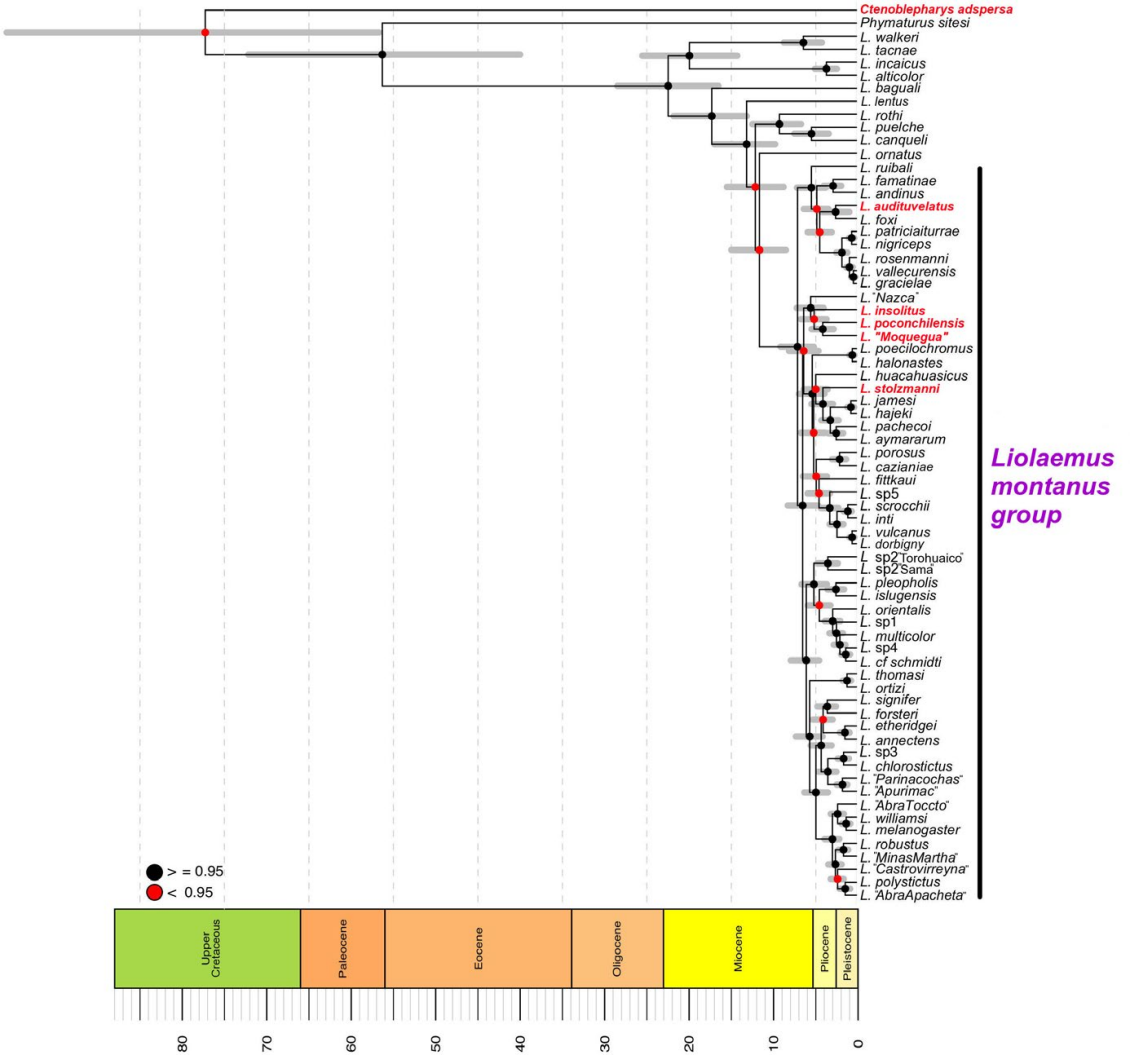
Divergence time tree including species of the *Liolaemus montanus* group and *Ctenoblepharys adspersa*. Putative convergent taxa are in red. Red and black dots on nodes indicate <0.95 and ≥0.95 posterior probabilities respectively. Grey bars are 95% confidence intervals for node ages

Main results of the Bayesian divergence time tree (BT; Figure 3) with posterior probabilities (PP) are shown, and differences with the maximum likelihood (ML) tree (Supporting Information Figure S2) are summarized below. Detailed results are given together with Supporting Information Figure S2. The BT tree shows a well‐supported (PP ≥0.95) *L. montanus* group and resolves the “Phrynosauroids” *L. poconchilensis*,* L. stolzmanni* and *L. audituvelatus* in three separate clades (Figure 3). The DT tree resolves ([*Liolaemus* + *P. sitesi*] + *C. adspersa*) with low support (PP ≤ 0.95), but in the ML tree these relationships are well‐supported.

The clade (*Liolaemus* + *P. sitesi*) diverged from *C. adspersa* ~77 million years ago (mya) in the Upper Cretaceous. The *L. montanus* group and *Liolaemus ornatus* (species representing the *Liolaemus darwini* group) diverged about 12 mya in the Miocene.

The clade (*L. insolitus* [*L. *“Moquegua” + *L. poconchilensis*]) has a mean age of 5 million years. The clade (*L. stolzmanni* [*Liolaemus pachecoi* + *Liolaemus aymararum*] [*Liolaemus jamesi* + *Liolaemus hajeki*]) has a mean age of 4.8 mya. The clade (*L. audituvelatus* + *Liolaemus foxi*) has a mean age of 2.5 mya. These three clades diverged between the end of the Miocene and the Pliocene.

### 3.2 Convergence analyses

**Figure 4:**
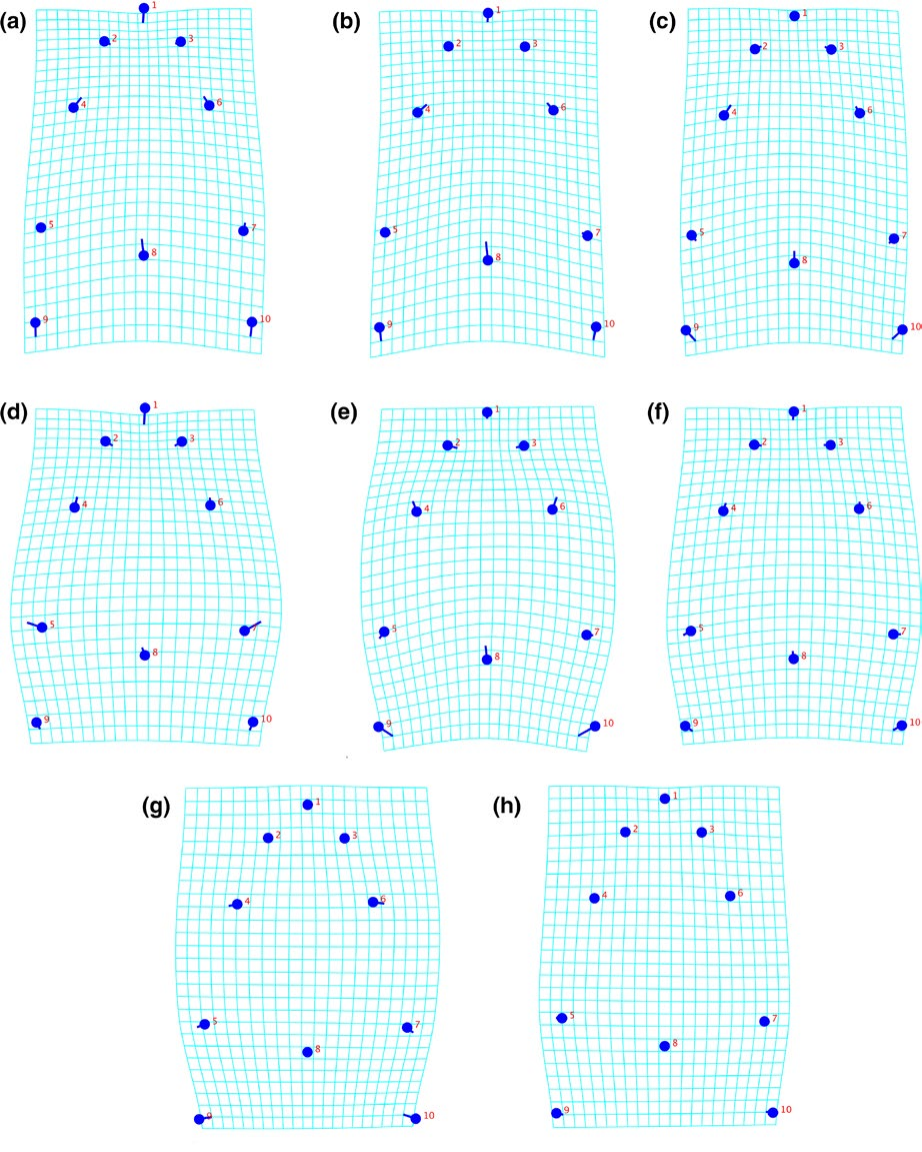
Transformation grids of head shape changes of each focal taxon relative to the reconstructed shape of their most recent common ancestor (MRCA). (a) MRCA of *Liolaemus audituvelatus*, (b) MRCA of *Liolaemus stolzmanni*, (c) *Liolaemus insolitus*, (d) *L. audituvelatus*, (e) *L. stolzmanni*, (f)* Liolaemus poconchilensis*, (g) *Ctenoblepharys adspersa*, (h) *Liolaemus* “Moquegua”

Reconstructed transformation grids of head shapes based on principal component scores and the pruned BT are shown in Figure 4. Differences of head shape between *L. audituvelatus* and its most recent common ancestor (MRCA; Figure 4a,d) are the medial displacements of landmarks 2–3, lateral displacements of landmarks 5 and 7, reduced displacement of landmark 8, and medial displacements of landmarks 9–10.

Differences between *L. stolzmanni* and its MRCA (Figure 4b,e) are the medial displacements of landmarks 2–3, lateral displacements of landmarks 4–7, reduced displacement of landmark 8, and medial displacements of landmarks 9–10.

Differences between *L. insolitus* (similar to its MRCA) and *L. *“Moquegua” + *L. poconchilensis* (Figure 4c,f,h) are the lateral displacements of landmarks 5 and 7, and reduced displacement of landmark 8.

*Ctenoblepharys adspersa* (Figure 4g) shows similar lateral displacements of landmarks 4–7 with *L. stolzmanni*, 5 and 7 with *L. *“Moquegua” + *L. poconchilensis*, and similar medial displacements of landmarks 9–10 with all focal taxa.

**Figure 5:**
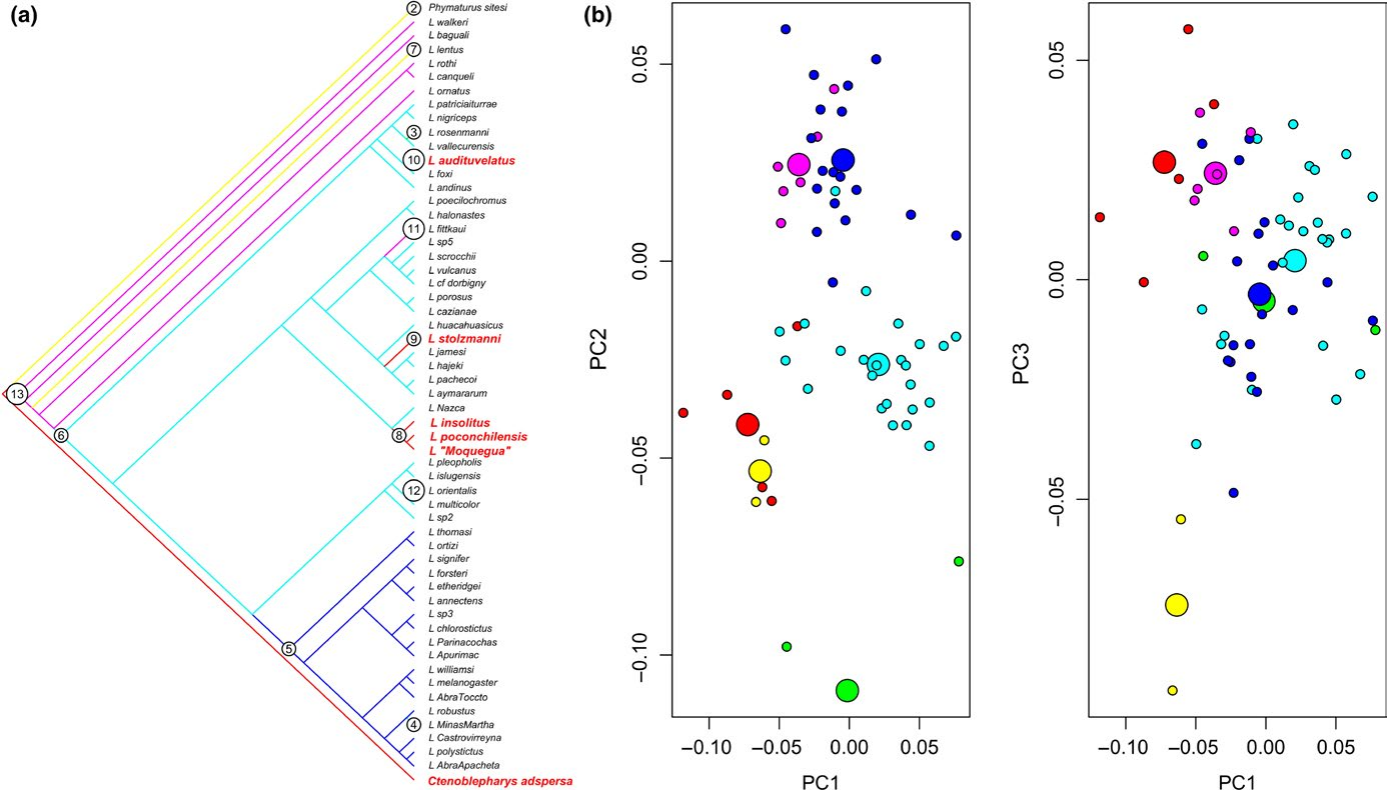
Results of SURFACE analysis. (a) Phylogeny showing placement of focal convergent taxa (colored in red). (b) Plot of trait values based on principal components (PC) 1 and PC2, and PC1 and PC3: small circles identify species and large circles are estimated adaptive optima; red circles identify species and estimated optima for the focal convergent regime

Our SURFACE analyses based on PC1–PC3 identify 13 phenotypic regimes of which all show convergence (Table 1; Figure 5a,b) and includes one convergent regime made by *C. adspersa*,* L. audituvelatus*,* L. poconchilensis*,* L. stolzmanni*,* L. insolitus* and *L. *“Moquegua” (in red; Figure 5a,b). The best model found by SURFACE (OUc; AICc = −756.2257) is an improvement over the multiple nonconvergent regime (OUnc; AIC = −717.4198), one peak (OU1; AIC = −677.58) and Brownian (BM; AIC = −649.3834) models. Other model parameters and regimes are shown in Table 1 and Figure 5.

**Table 1:** Surface analysis parameters for different models of evolution: OU_nc_ (non‐convergent peak model), OU_c_ (model with convergent adaptive peak), OU_1_ (model with one adaptive peak) and BM (Brownian motion model)

Parameters	Models			BM
	OU_c_	OU_nc_	OU_1_	
*k* (number of regime shifts)	13	13	1	0
*K*′ (number of distinct regimes)	6	13	1	0
Δ*k* (*k* − *k*′)	7	0	0	0
*c* (number of shifts that are toward convergent regimes occupied by multiple lineages)	13	0	0	0
*k*′_conv	6	0	0	0
*k*′_nonconv	0	13	1	0
AIC_c_	−756.23	−717.42	−677.58	−649.38

**Figure 6:**
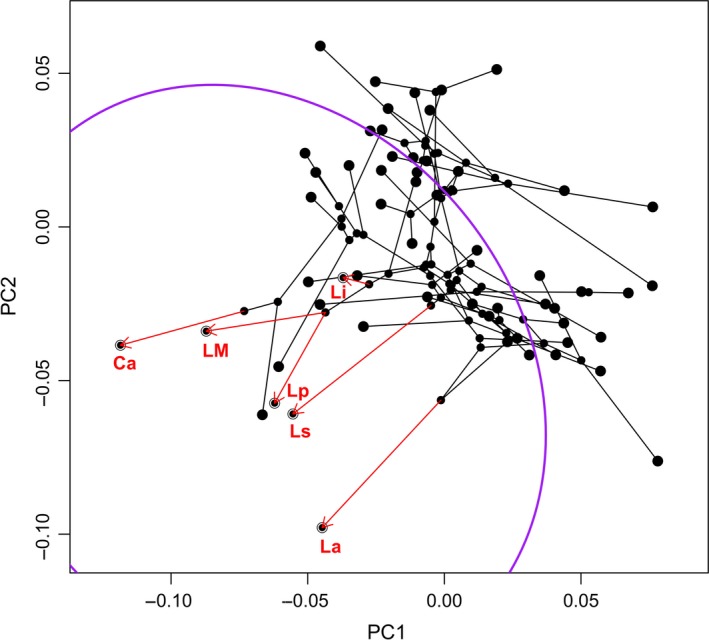
CONVEVOL phylomorphospace of 57 species in principal components (PC) 1 and PC2. The black lines connect only non‐focal species and red arrows connect nodes to six focal convergent species. *Ctenoblepharys adspersa* (Ca), *Liolaemus audituvelatus* (La), *Liolaemus insolitus* (Li), *Liolaemus poconchilensis* (Lp), *Liolaemus stolzmanni* (Ls) and *Liolaemus* “Moquegua” (LM). The violet curve defines a distinct region of the phylomorphospace where these six taxa are present

C1–C5 metrics of convergences based on PC1–PC3 are shown in Table 2. C1–C4 values were significantly higher for *C. adspersa*,* L. audituvelatus*,* L. insolitus*,* L. poconchilensis*,* L. stolzmanni* and *L. *“Moquegua” than the other subsets (Table 2). The C5 metric shows that *C. adspersa*,* L. audituvelatus*,* L. insolitus*,* L. poconchilensis*,* L. stolzmanni* and *L. *“Moquegua” and the two subsets (Table 2) significantly cluster in a distinct region of the phylomorphospace (Table 2, Figure 6).

**Table 2:** CONVEVOL and WHEATSHEAF results

Convergent taxa	C1	C2	C3	C4	C5
*Ctenoblepharys adspersa*,* Liolaemus audituvelatus*,* Liolaemus poconchilensis*,* Liolaemus stolzmanni*	0.39141363 (0.005)	0.04812552 (0.000)	0.01076006 (0.0998)	0.01795421 (0.013972)	4 (0.012)
	**Wheatsheaf Index: 1.404266**	**Lower 95% CI: 1.378**	**Upper 95% CI: 1.698**	***p*‐value: 0.139**	
*Ctenoblepharys adspersa*,* Liolaemus audituvelatus*,* Liolaemus poconchilensis*,* Liolaemus stolzmanni*,* Liolaemus* "*Moquegua*"	0.45562096 (0.001)	0.05638699 (0.000)	0.01260718 (0.013)	0.02103632 (0.002)	5 (0.008)
	**Wheatsheaf Index: 1.272332**	**Lower 95% CI: 1.248581**	**Upper 95% CI: 1.462739**	***p*‐value: 0.115**	
*Ctenoblepharys adspersa*,* Liolaemus audituvelatus*,* Liolaemus poconchilensis*,* Liolaemus stolzmanni*,* Liolaemus* "*Moquegua*", *Liolaemus insolitus*	0.46254834 (0.000)	0.05846191 (0.000)	0.0130711 (0.014)	0.02181041 (0.000)	6 (0.024)
	**Wheatsheaf Index: 1.030869**	**Lower 95% CI: 1.009809**	**Upper 95% CI: 1.196133**	***p*‐value: 0.512**	

Notes

C1–C5 convergence metrics derived from CONVEVOL analyses. High numbers in C1‐C4 indicates strong convergence and C5 shows the number of convergent focal taxa that occupy a distinct region in phylomorphospace. Numbers in parenthesis are *p*‐values. In bold results of WHEATSHEAF index. Higher values of this index indicate that the convergent taxa are more similar to each other than non‐focal taxa.

CI: confidence interval.

The WHEATSHEAF index was higher for the subset *C. adspersa*,* L. audituvelatus*,* L. poconchilensis* and *L. stolzmanni* (Table 2) than for the other subset and all focal taxa. However, results were not significant for all of them.

## 4 DISCUSSION

Convergence might be due to chance, constraints or natural selection (Losos, 2011; Mahler, Weber, Wagner, & Ingram, 2017). Adaptive convergence implies that natural selection has produced the same phenotype in similar environments in unrelated taxa (Losos, 2011).

Constraints (developmental and phylogenetic) imposed by ancestors are enforced by natural selection and thus by the circumstances under which organisms develop and evolve (Vermeij, 2015). Natural selection and constraint are probably more important than historical contingency in explaining the repeated adaptations to the same habitat in phylogenetically close taxa (Hagman & Ord, 2016). In contrast, historical contingency may be more important in producing unique adaptations in phylogenetically distant taxa (Hagman & Ord, 2016). Natural selection is thought to erase the stamp of historical contingency when comparing species of the same genus or populations within a species, but not so in highly divergent taxa (Ord & Summers, 2015). In addition, evolutionary paths that converge on a local fitness optimum over a short time period will not necessarily achieve the highest adaptive peak over a longer period because of stochastic events (Orgogozo, 2015).

At least one of the three methods applied in our study, SURFACE, is based on the assumption that convergence is adaptive (Arbuckle et al., 2014; Ingram & Mahler, 2013). SURFACE employs the metaphor of Wright and Simpson that evolution is a local search by fitter geno/phenotypes climbing higher peaks in an adaptive landscape (Arnold, Pfrender, & Jones, 2001; Niklas, 1995). This method identifies convergent taxa via evolutionary models and results are interpreted as occupation of the same or very similar adaptive peaks, which is then taken to characterize a selective regime (Ingram & Mahler, 2013; Mahler et al., 2017). In contrast, the C1–C5 metrics test whether convergence in putative taxa is different from chance without invoking any evolutionary process a priori (Stayton, 2015a, 2015b ). However, there is still controversy about whether phylogenetic comparative models used to identify and measure convergence, are or are not process free (Mahler et al., 2017; Stayton, 2015b).

Our SURFACE analysis identified 13 convergent peaks and one of them reached by our six species focal species: *C. adspersa*,* L. audituvelatus*,* L. insolitus*,* L. poconchilensis*,* L. stolzmanni* and *L. *“Moquegua” (Figure 5; Table 1). Convergence of these six taxa is strongly supported by higher C1–C4 metrics, in comparison with a subset including only taxa with highly modified head shape (*L. insolitus* excluded) and another including only taxa with serrate combs in the eyes (*L. insolitus* and *L. *“Moquegua” excluded; Table 2). Both results suggest a significant case convergence for these Pacific desert species reaching the same adaptive peak.

Convergent *Liolaemus* and *C. adspersa* show a lateral widening, and posteromedial stretching of the head (Figure 4c–h). Assuming evolutionary modifications in head shape in these convergent taxa are related to a loss of bite force and reduction in the morphology of prey‐processing features, this reduction in traits may be a response of disuse driven by natural selection (Fong, Kane, & Culver, 1995; Porter & Crandall, 2003). The benefit of an unused character may be outweighed by its fitness cost in the current environment, but reduction of an unused trait may be associated with increased fitness (Hall & Colegrave, 2008). In environments with low resources, the fitness cost imposed by an unused trait is greatest (Hall & Colegrave, 2008). Probably in the low resource environments of the Peruvian and Atacama deserts, convergent lizards with reduced unused traits may have higher fitness.

WHEATSHEAF index was higher for the subset *C. adspersa*,* L. audituvelatus*,* L. poconchilensis* and *L. stolzmanni* than for the complete set of convergent taxa. However, results were not significant in all cases (Table 2), suggesting that the phenotypic similarities among these species do not qualify as especially strong convergence. *Ctenoblepharys adspersa* and all convergent Pacific desert *Liolaemus* are morphologically similar in head shape, but not identical (Figure 4c–h). In addition, Pacific desert *Liolaemus* are not phylogenetically distant between each other (Figure 3).

The origin of the hyper‐aridity in the Peruvian and Atacama deserts (~25 My) is consistent with the ages of focal *Liolaemus* clades in our time‐calibrated tree (Figure 3; Rundel et al., 2007). In addition, our BT resolves the origin of this head shape first in *C. adspersa* and three times in the *L. montanus* group (Figure 5). This suggests that, in addition to selection, phylogenetic constraint may have also been involved in producing the same phenotype in these desert lizards. However, information is lacking on whether similar or identical mechanisms (e.g. same developmental pathway, same genes, or same specific‐site mutation in the same genes) have been responsible for the independent origin of these same phenotypic traits. Results of such research would clarify the role of constraint in the evolution of adaptation (Agrawal, 2017; Arendt & Reznick, 2007).

Our results do suggest, however, that despite its deep divergence and thus potential exposure to different contingent factors, natural selection has been stronger than historical contingency in producing the same convergent traits in the phylogenetically distant *C. adspersa* and younger species of the *L. montanus* group (for traits examined in this study). However, historical contingencies probably were also important when considering these traits at higher taxonomic levels. For instance, the convergent taxa in our study have been hypothesized to be phenotypically similar to the phylogenetically very distantly related lizard *Phrynocephalus* (family Agamidae; Valladares, 2004), suggesting than a toad‐like head may have been present in an older lizard ancestor (~168 My; Zheng & Wiens, 2016). Although a formal test of convergence is needed, both lizard families have probably been exposed to different contingent events and may also have unique adaptations for living in different desert environments (Arnold, 1995; Melville, Harmon, & Losos, 2006).

## CONFLICT OF INTEREST

None declared.

## AUTHORS’ CONTRIBUTIONS

CA provided substantial contributions to the conception or design of the work; the acquisition, analysis, and interpretation of data; drafting and final approval of the version to be published; agreement to be accountable for all aspects of the work in ensuring that questions related to the accuracy or integrity of any part of the manuscript are appropriately investigated and resolved. LA, IDR, LJ, MM, JTP, PLW, and JWS revised the manuscript critically for important intellectual content and final approval of the version to be published. LA, IDL, LJ, MM, JTP, and JWS provided acquisition of data for the work. PLW provided substantial contributions to analysis, and interpretation of data for the work.

## DATA ACCESSIBILITY

New sequences were deposited in GenBank and their numbers are given in Supporting Information Appendix S2.
