# Supplementary material for: The shadow of the past: Convergence of young and old South American desert lizards as measured by head shape traits
Source: Ecol Evol. 2018 Nov 26;8(23):11399–409. doi: 10.1002/ece3.4548 (PMC6303702; doi:10.1002/ece3.4548)

**Supplementary Figures**

Supplementary Figure 1. Maximum likelihood multilocus tree based on 181 terminals. Candidate species of the *Liolaemus montanus* group are highlighted in yellow and selected individuals for phylogenetic analyses are highlighted in red (see below). Asterisks show bootstrap support ≥ 70%.


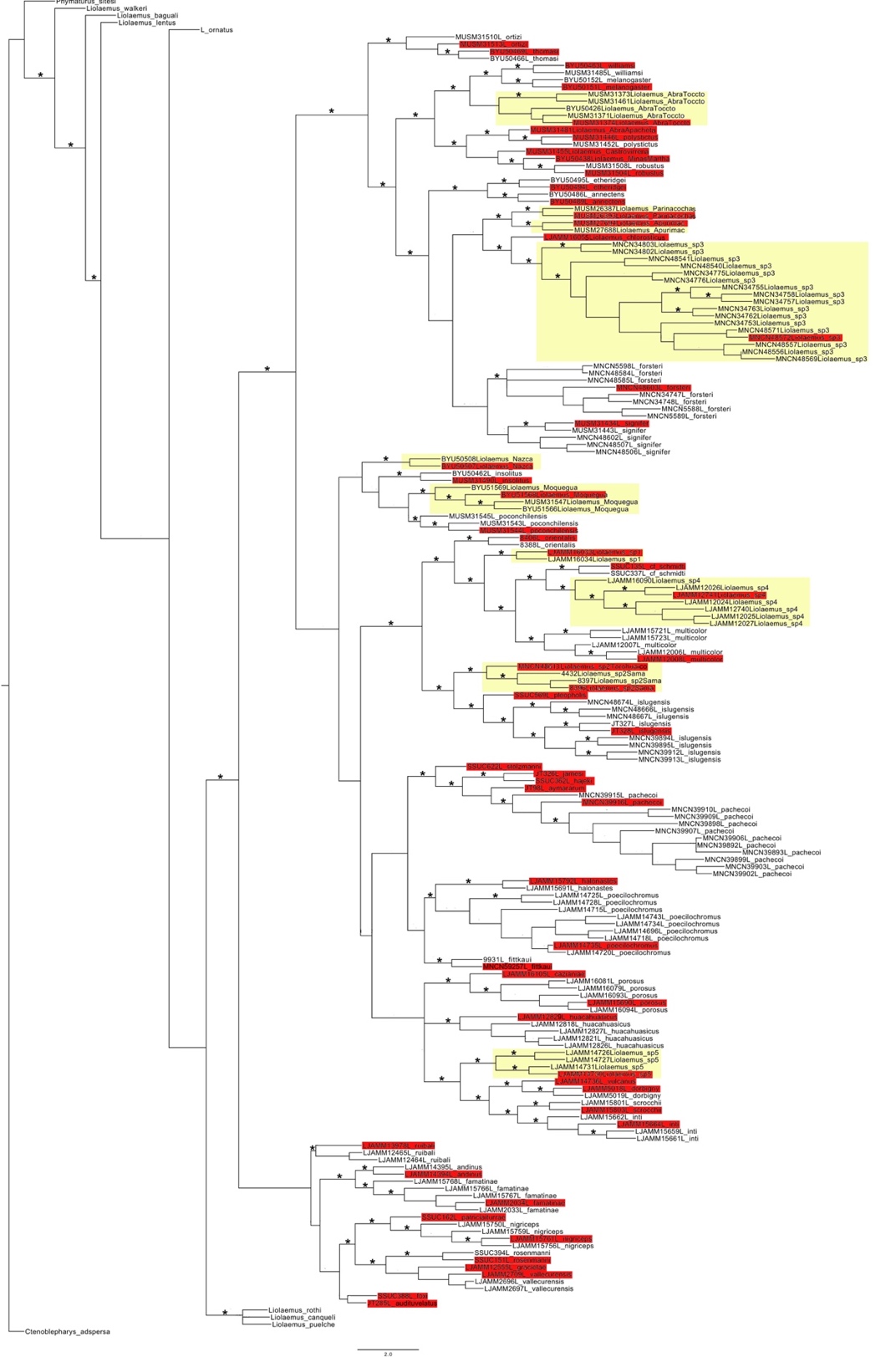


Comparison between bayesian and maximum likelihood trees

Differences between the Bayesian divergence time tree (DT; Fig. 3) and maximum likelihood (ML) tree (Supplementary Figure 2 [Fig. S2]) are summarized below. The DT shows a well-supported (PP ≥ 0.95) *Liolaemus* *montanus* group (Fig 3), which includes two major clades; one with high support and formed by *L. ruibali*, and sister to a group with low support (PP < 0.95), which is composed of two small clades. One of these (*L. famatinae* + *L. andinus*) is inferred with high support (PP ≥ 0.95), and another with low support; this last one includes (*L. manueli* + *L. foxi*) with PP ≥ 0.95. The clade ((*L.* *patriciaiturrae* + *L. nigriceps*) (*L. rosenmanni* (*L. gracielae* + *L. vallecurensis*)) and all nested clades have strong support (PP ≥ 0.95). In the ML tree this major clade including *L. ruibali* and all nested clades is weakly supported (BS ≤ 70).

This major clade is sister to a well-supported (PP ≥ 0.95) group that in turn includes two nested clades. The most inclusive one, (*Liolaemus* “Nazca”(*L. insolitus* (*L. poconchilensis* + *Liolaemus* “Moquegua”))) is well supported (PP ≥ 0.95), while the nested clade (*L. insolitus* (*L. poconchilensis* + *Liolaemus* “Moquegua”)) has PP < 0.95, and the nested clade within the latter (*L. poconchilensis* + *Liolaemus* “Moquegua”) is also strongly supported (PP ≥ 0.95). The ML tree infers a weakly supported clade (*L. insolitus* (*L.* “Nazca” (*L. poconchilensis* + *Liolaemus* “Moquegua”))) (BS ≤ 70), and this clade is not nested within one of the two big clades as in DT tree, but it is recovered as a separate group (Fig. S2).

This small clade is sister to a large monophyletic group (PP ≥ 0.95) composed of (*Liolaemus poecilochromus* + *L. halonastes*) with PP ≥ 0.95. A separate weakly supported clade (PP < 0.95) resolves *L. huacahuasicus* as the sister group to a well-supported clade (PP ≥ 0.95) with the following structure: (*L. stolzmanni* + ((*L. hajeki*, *L. jamesi*) + (*L. aymararum* + *L. pachecoi*))); all nested subclades also have high support. The second clade has low support and is composed of (*L. cazianiae* + *L. porosus*) with PP ≥ 0.95, and sister to a group with low support formed by *L. fittkaui* and (*L.* sp. 5. ((*L. inti* + *L. scrocchii*) (*L. vulcanus* + *L.* *dorbigny*))). This second clade is strongly supported, as are all subclades nested within it (PP ≥ 0.95). The ML topology differs in that it recovers *L. huacahuasicus* with weak support (BS ≤ 70) as sister to (*L. cazianae* + *L. porosus*) (Fig. S2).

The other big clade is well supported (PP ≥ 0.95), and is composed by *L.* sp. 2 and a clade with low support, with groups (*L. islugensis + L. pleopholis*) with PP ≥ 0.95, and (*L. orientalis (L. sp.* 1 (*L. multicolor* (*L.* sp. 4 + *L.* cf. *schmidti*)))), with high support, and as well as all other nested groups. The ML tree differs in resolving several clades with low support (BS ≤ 70) (Fig. S2).

The other major clade is also well-supported (PP ≥ 0.95) and formed by (*L. ortizi* + *L. thomasi*) with PP ≥ 0.95, and a large clade with high support and composed of two groups. One of them is well supported, having a clade ((*L. forsteri* + *L. signifer*) (*L. annectens* + *L. etheridgei*)) with low support (PP < 0.95) but with nested clades having high support; and ((*L.* sp. 3 + *L. chlorostictus*) (*L.* “Parinacochas” + *L.* “Apurimac”)) having high support including all nested clades. The other group is also well supported and is composed of a clade (*L.* “AbraToccto” (*L. williamsi* + *L. melanogaster*)) with PP ≥ 0.95, and another clade also well-supported (*L.* “MinasMartha” + *L. robustus)* and (*L.* “Castrovirreyna” (*L. polystictus* + *L.* “AbraApacheta”) with PP < 0.95, but (*L. polystictus* + *L.* “AbraApacheta”) having PP ≥ 0.95. The ML tree differs only in the position of *L. forsteri* but with low support (BS ≤ 70).

Supplementary Figure 2. Maximum likelihood multilocus tree showing relationships between species and candidate species of the *Liolaemus montanus* group, representatives of other species groups in *Liolaemus*, other genera in Liolaemiidae and outgroups. Asterisks show bootstrap support ≥ 70%. Terminals in blue are putative convergent forms in head morphology.


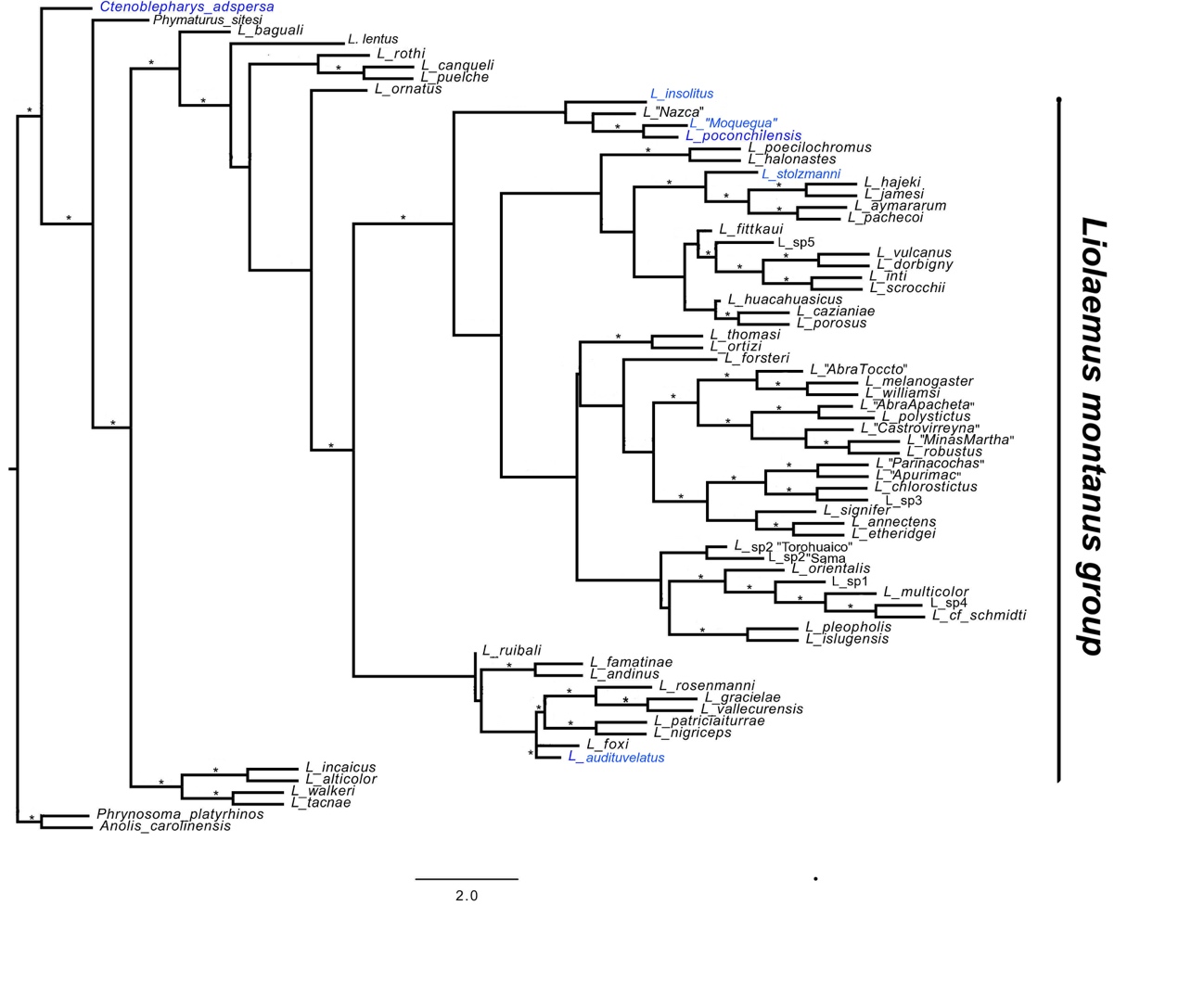

Supplement: Supplementary file 1 [file ECE3-8-11399-s001.docx]
