# Supplementary material for: The shadow of the past: Convergence of young and old South American desert lizards as measured by head shape traits
Source: Ecol Evol. 2018 Nov 26;8(23):11399–409. doi: 10.1002/ece3.4548 (PMC6303702; doi:10.1002/ece3.4548)
Supplement: Supplementary file 2 [file ECE3-8-11399-s002.docx]

**Supplementary Material 1.**

Sampling, DNA extraction, amplification and sequencing

Lizards were collected during the day by hand, photographed and euthanized with an injection of sodium pentobarbital. Tissue samples were collected in duplicate, stored in 96% ethanol and deposited at the M. L. Bean Life Science Museum at Brigham Young University (BYU) and Museo de Historia Natural de San Marcos (MUSM) in Lima, Perú, and voucher specimens were shared between these two institutions on a 50:50 basis. Whole specimens were fixed in 10% formalin, and transferred to 70% ethanol for permanent storage in the aforementioned museum collections. See Table S2 for locality details.

Total genomic DNA was extracted from liver/muscle tissue using the animal tissue extraction protocol in the Qiagen protocol (Qiagen, Inc., Valencia, CA). The mitochondrial cyt-b gene (652 bp) was sequenced for all individuals, and non-redundant haplotypes were sequenced for the mtDNA 12S region (~788 bp), and five nuclear gene regions: CMOS (398 bp), EXPH5 (888 bp), KIF24 (478 bp), MXRA5 (776 bp), and PRLR (~534 bp). Double stranded polymerase chain reactions (PCR) amplified target regions under the conditions described in Aguilar et al. (2017). Primer information is shown in Table S1. PCR products were visualized on 1% agarose gels to ensure the targeted products were cleanly amplified, then purified using a MultiScreen PCR (mu) 96 (Millipore Corp., Billerica, MA), and directly sequenced using the BigDye Terminator v 3.1 Cycle Sequencing Ready Reaction (Applied Biosystems, Foster City, CA). The cycle sequencing reactions were purified using Sephadex G-50 Fine (GE Healthcare) and MultiScreen HV plates (Millipore Corp.). Samples were then analyzed on an ABI3730xl DNA Analyzer in the BYU DNA Sequencing Center.

Taxon sampling for phylogenetic inference

Some individuals of the *Liolaemus* *montanus* group could not be assigned to any known species because they are juveniles or females lacking diagnostic morphological features, or they may represent new species. All individuals sequenced in this study, along with their taxonomic assignments are summarized in Table S1. To resolve taxonomic uncertainties, we implemented a maximum likelihood (ML) concatenated phylogenetic analysis (see below for details) of the mitochondrial and nuclear data using all individuals (181 terminals, SM 1). Individuals not assigned to any known species of the *L. montanus* group and forming clades with high bootstrap support (BS ≥ 70) (Hillis and Bull 1993; with caveats) were considered candidate species and are shown in Supplementary Figures (SF 1). We subsampled the concatenated tree by selecting single individuals representing either species or candidate species of the *L.* *montanus* group for further analyses.

Our analyses included 43 taxa assigned to the *Liolaemus* *montanus* group (Lobo et al., 2010) and 13 candidate species (see above, and Aguilar et al., 2017). We also included *Liolaemus lentus* (*anomalus* group), *L. puelche* Avila et al., 2007, *L. canqueli* Cei,1975, *L. ornatus* Koslowsky, 1898, *L. rothi* Koslowsky, 1898 and *L. baguali* Cei & Scolaro, 1983 (representing other species groups in the subgenus *Eulaemus*)*, L. walkeri* Shreve, 1938*, L. tacnae* (Shreve, 1941)*, L. alticolor* Barbour, 1909*, L. incaicus* Lobo et al., 2007 (subgenus *Liolaemus*), *Phymaturus sitesi* Avila et al., 2011, *Ctenoblepharys adspersa* (Liolaemidae), *Anolis carolinensis* Voigt, 1832 (Dactyloidae) and *Phrynosoma platyrhinos* Girard, 1852 (Phrynosomatidae). These last two taxa were selected as the outgroup (Wiens et al., 2012). For some of these taxa, homologous regions were obtained from GenBank (SM 2). New sequences were deposited in GenBank and their numbers are given in SM 2.

Table S1. Molecular markers and primers used in this study

| Locus | Genome | Substitution model | Primers | Reference |
| --- | --- | --- | --- | --- |
| CYTB | mtDNA | HKY+G | IguaF2, IguaR2 | Corl et al. (2010) |
| 12S | mtDNA | TrN+I+G | tphe, E | Wiens et al. (1999) |
| CMOS | nuclear | K80+G | FU-F, FU-R | Wiens et al. (1999) |
| EXPH5 | nuclear | HKY | F1, R1 | Portik et al. (2012) |
| KIF24 | nuclear | HKY | F1, R2 | Portik et al. (2012) |
| MXRA5 | nuclear | HKY | F, R | Portik et al. (2012) |
| PRLR | nuclear | HKY+G | F1, R3 | Townsend et al. (2008) |

Table S2. Specimens sequenced for this study, their museum or field numbers, and localities. mncn numbers are dna museum numbers.

|  | Museum/ Field Acronym | Number | Country, Region, Locality | Genus | Species |
| --- | --- | --- | --- | --- | --- |
| 1 | LJAMM-CNP | 5019 | Argentina, Catamarca, Belén | Liolaemus | dorbigny |
| 2 | LJAMM-CNP | 5018 | Argentina, Catamarca, Belén | Liolaemus | dorbigny |
| 3 | LJAMM-CNP | 14395 | Argentina, Catamarca, Tigonasta | Liolaemus | andinus |
| 4 | LJAMM-CNP | 14394 | Argentina, Catamarca, Tigonasta | Liolaemus | andinus |
| 5 | LJAMM-CNP | 16105 | Argentina, Salta, Los Andes | Liolaemus | cazianae |
| 6 | LJAMM-CNP | 14735 | Argentina, Catamarca, Antofagasta de la Sierra | Liolaemus | poecilochromus |
| 7 | LJAMM-CNP | 14725 | Argentina, Catamarca, Antofagasta de la Sierra | Liolaemus | poecilochromus |
| 8 | LJAMM-CNP | 14720 | Argentina, Catamarca, Antofagasta de la Sierra | Liolaemus | poecilochromus |
| 9 | LJAMM-CNP | 14734 | Argentina, Catamarca, Antofagasta de la Sierra | Liolaemus | poecilochromus |
| 10 | LJAMM-CNP | 14728 | Argentina, Catamarca, Antofagasta de la Sierra | Liolaemus | poecilochromus |
| 11 | LJAMM-CNP | 14743 | Argentina, Catamarca, Antofagasta de la Sierra | Liolaemus | poecilochromus |
| 12 | LJAMM-CNP | 14715 | Argentina, Catamarca, Antofagasta de la Sierra | Liolaemus | poecilochromus |
| 13 | LJAMM-CNP | 14718 | Argentina, Catamarca, Antofagasta de la Sierra | Liolaemus | poecilochromus |
| 14 | LJAMM-CNP | 14696 | Argentina, Catamarca, Antofagasta de la Sierra | Liolaemus | poecilochromus |
| 15 | LJAMM-CNP | 14730 | Argentina, Catamarca, Antofagasta de la Sierra | Liolaemus | sp5 |
| 16 | LJAMM-CNP | 14731 | Argentina, Catamarca, Antofagasta de la Sierra | Liolaemus | sp5 |
| 17 | LJAMM-CNP | 14727 | Argentina, Salta, Los Andes | Liolaemus | sp5 |
| 18 | LJAMM-CNP | 14726 | Argentina, Salta, Los Andes | Liolaemus | sp5 |
| 19 | LJAMM-CNP | 15767 | Argentina, La Rioja, Famatina | Liolaemus | famatinae |
| 20 | LJAMM-CNP | 2034 | Argentina, La Rioja, Famatina | Liolaemus | famatinae |
| 21 | LJAMM-CNP | 15768 | Argentina, La Rioja, Famatina | Liolaemus | famatinae |
| 22 | LJAMM-CNP | 15766 | Argentina, La Rioja, Famatina | Liolaemus | famatinae |
| 23 | LJAMM-CNP | 2033 | Argentina, La Rioja, Famatina | Liolaemus | famatinae |
| 24 | LJAMM-CNP | 12555 | Argentina, La Rioja, Vinchina | Liolaemus | gracielae |
| 25 | LJAMM-CNP | 12008 | Argentina, Jujuy, Cochinoca | Liolaemus | multicolor |
| 26 | LJAMM-CNP | 12007 | Argentina, Jujuy, Cochinoca | Liolaemus | multicolor |
| 27 | LJAMM-CNP | 12006 | Argentina, Jujuy, Cochinoca | Liolaemus | multicolor |
| 28 | LJAMM-CNP | 15721 | Argentina, Salta, La Poma | Liolaemus | multicolor |
| 29 | LJAMM-CNP | 15723 | Argentina, Salta, La Poma | Liolaemus | multicolor |
| 30 | LJAMM-CNP | 12465 | Argentina, Mendoza, Las Heras | Liolaemus | ruibali |
| 31 | LJAMM-CNP | 13978 | Argentina, Mendoza, Las Heras | Liolaemus | ruibali |
| 32 | LJAMM-CNP | 12464 | Argentina, Mendoza, Las Heras | Liolaemus | ruibali |
| 33 | LJAMM-CNP | 15690 | Argentina, Salta, Los Andes | Liolaemus | porosus |
| 34 | LJAMM-CNP | 16081 | Argentina, Salta, Los Andes | Liolaemus | porosus |
| 35 | LJAMM-CNP | 16093 | Argentina, Salta, Los Andes | Liolaemus | porosus |
| 36 | LJAMM-CNP | 16094 | Argentina, Salta, Los Andes | Liolaemus | porosus |
| 37 | LJAMM-CNP | 16079 | Argentina, Salta, Los Andes | Liolaemus | porosus |
| 38 | LJAMM-CNP | 15756 | Argentina, Salta, Los Andes | Liolaemus | nigriceps |
| 39 | LJAMM-CNP | 15759 | Argentina, Salta, Los Andes | Liolaemus | nigriceps |
| 40 | LJAMM-CNP | 15750 | Argentina, Salta, Los Andes | Liolaemus | nigriceps |
| 41 | LJAMM-CNP | 15761 | Argentina, Salta, Los Andes | Liolaemus | nigriceps |
| 42 | LJAMM-CNP | 12826 | Argentina, Tucuman, Trancas | Liolaemus | huacahuasicus |
| 43 | LJAMM-CNP | 12827 | Argentina, Tucuman, Trancas | Liolaemus | huacahuasicus |
| 44 | LJAMM-CNP | 12828 | Argentina, Tucuman, Trancas | Liolaemus | huacahuasicus |
| 45 | LJAMM-CNP | 12818 | Argentina, Tucuman, Trancas | Liolaemus | huacahuasicus |
| 46 | LJAMM-CNP | 12821 | Argentina, Tucuman, Trancas | Liolaemus | huacahuasicus |
| 47 | LJAMM-CNP | 16054 | Argentina, Jujuy, Rinconada | Liolaemus | chlorostictus |
| 48 | LJAMM-CNP | 15662 | Argentina, Salta, Cachi | Liolaemus | inti |
| 49 | LJAMM-CNP | 15664 | Argentina, Salta, Cachi | Liolaemus | inti |
| 50 | LJAMM-CNP | 15659 | Argentina, Salta, Cachi | Liolaemus | inti |
| 51 | LJAMM-CNP | 15661 | Argentina, Salta, Cachi | Liolaemus | inti |
| 52 | LJAMM-CNP | 16034 | Argentina, Jujuy, Humahuaca | Liolaemus | sp.1 |
| 53 | LJAMM-CNP | 16033 | Argentina, Jujuy, Humahuaca | Liolaemus | sp.1 |
| 54 | LJAMM-CNP | 12025 | Argentina, Salta, Rosario de Lerma | Liolaemus | sp4 |
| 55 | LJAMM-CNP | 16090 | Argentina, Jujuy, Susques | Liolaemus | sp4 |
| 56 | LJAMM-CNP | 12024 | Argentina, Salta, Rosario de Lerma | Liolaemus | sp4 |
| 57 | LJAMM-CNP | 12026 | Argentina, Salta, Rosario de Lerma | Liolaemus | sp4 |
| 58 | LJAMM-CNP | 12027 | Argentina, Salta, Rosario de Lerma | Liolaemus | sp4 |
| 59 | LJAMM-CNP | 12740 | Argentina, Salta, San Antonio de los Cobres | Liolaemus | sp4 |
| 60 | LJAMM-CNP | 12741 | Argentina, Salta, San Antonio de los Cobres | Liolaemus | sp4 |
| 61 | LJAMM-CNP | 15691 | Argentina, Salta, Los Andes | Liolaemus | halonastes |
| 62 | LJAMM-CNP | 15792 | Argentina, Salta, Los Andes | Liolaemus | halonastes |
| 63 | LJAMM-CNP | 15803 | Argentina, Jujuy, Susques | Liolaemus | scrocchii |
| 64 | LJAMM-CNP | 15801 | Argentina, Jujuy, Susques | Liolaemus | scrocchii |
| 65 | LJAMM-CNP | 2709 | Argentina, San Juan, Iglesia | Liolaemus | vallecurensis |
| 66 | LJAMM-CNP | 2696 | Argentina, San Juan, Iglesia | Liolaemus | vallecurensis |
| 67 | LJAMM-CNP | 2697 | Argentina, San Juan, Iglesia | Liolaemus | vallecurensis |
| 68 | LJAMM-CNP | 14736 | Argentina, Catamarca, Antofagasta de la Sierra | Liolaemus | vulcanus |
| 69 | LJAMM-CNP | 12190 | Argentina | Phrymaturus | sitesi |
| 70 | MNCN | 48614 | Bolivia, Tarija, Torohuaico | Liolaemus | sp.2"Torohuaico" |
| 71 | No assigned | 4432 | Bolivia, Sama | Liolaemus | sp.2-"Sama" |
| 72 | No assigned | 8397 | Bolivia, Tarija, Serranía de Sama. Avilez. | Liolaemus | sp.2-"Sama" |
| 73 | No assigned | 8396 | Bolivia, Tarija, Serranía de Sama. Avilez. | Liolaemus | sp.2-"Sama" |
| 74 | No assigned | 8406 | Bolivia, Tarija, Serranía de Sama. Avilez. | Liolaemus | orientalis |
| 75 | MNCN | 48604 | Bolivia, La Paz, Camino a montañas en Millipaya. Sorata | Liolaemus | orientalis |
| 76 |  | 9931 | Bolivia | Liolaemus | fittkaui |
| 77 | MNCN | 59257 | Bolivia, Cochabamba, Cerca de Laguna Coaricocha | Liolaemus | fittkaui |
| 78 | MNCN | 34755 | Bolivia, La Paz, Entre 5-8 km al sur de Charaña en dirección a Achuta | Liolaemus | sp.3 |
| 79 | MNCN | 34757 | Bolivia, La Paz, Río Casapilla, antes del desvío a Khauniri. Provincia Pacajes | Liolaemus | sp.3 |
| 80 | MNCN | 34758 | Bolivia, La Paz, Río Casapilla, antes del desvío a Khauniri. Provincia Pacajes | Liolaemus | sp.3 |
| 81 | MNCN | 34762 | Bolivia, Oruro, Parque Nacional Sajama. Provincia Sajama | Liolaemus | sp.3 |
| 82 | MNCN | 34763 | Bolivia, Oruro, Parque Nacional Sajama. Provincia Sajama | Liolaemus | sp.3 |
| 83 | MNCN | 34775 | Bolivia, Oruro, Entre Curahuara de Carangas y Torora. Provincia Carangas | Liolaemus | sp.3 |
| 84 | MNCN | 34776 | Bolivia, Oruro, Entre Curahuara de Carangas y Torora. Provincia Carangas | Liolaemus | sp.3 |
| 85 | MNCN | 34753 | Bolivia, La Paz, Carretera entre Viacha y Charaña. Provincia Pacajes | Liolaemus | sp.3 |
| 86 | MNCN | 34803 | Bolivia, Cochabamba, Camino a Misicuni. Provincia Quillacollo | Liolaemus | sp.3 |
| 87 | MNCN | 34802 | Bolivia, Cochabamba, Camino a Misicuni. Provincia Quillacollo | Liolaemus | sp.3 |
| 88 | MNCN | 48540 | Bolivia, Cochabamba, La Cumbre. Carretera entre Oruro y Cochabamba | Liolaemus | sp.3 |
| 89 | MNCN | 48541 | Bolivia, Cochabamba, La Cumbre. Carretera entre Oruro y Cochabamba | Liolaemus | sp.3 |
| 90 | MNCN | 48556 | Bolivia, Oruro, Camino entre Colquiri y Cabari | Liolaemus | sp.3 |
| 91 | MNCN | 48557 | Bolivia, Oruro, Camino entre Colquiri y Cabari | Liolaemus | sp.3 |
| 92 | MNCN | 48569 | Bolivia, La Paz, Camino entre Caxata y Conani. Loaiza | Liolaemus | sp.3 |
| 93 | MNCN | 48571 | Bolivia, La Paz, Camino entre Caxata y Conani. Loaiza | Liolaemus | sp.3 |
| 94 | MNCN | 48572 | Bolivia, La Paz, Camino entre Caxata y Conani. Loaiza | Liolaemus | sp.3 |
| 95 | MNCN | 39892 | Bolivia, Potosí, Isla, Salar de Uyuni, cerca de Cerro Pabellón. Daniel Campos | Liolaemus | pachecoi |
| 96 | MNCN | 39893 | Bolivia, Potosí, Isla, Salar de Uyuni, cerca de Cerro Pabellón. Daniel Campos | Liolaemus | pachecoi |
| 97 | MNCN | 39898 | Bolivia, Potosí, Isla, Isla de Cerro Pabellón. Salar de Uyuni. Daniel Campos | Liolaemus | pachecoi |
| 98 | MNCN | 39899 | Bolivia, Potosí, Isla, Isla de Cerro Pabellón. Salar de Uyuni. Daniel Campos | Liolaemus | pachecoi |
| 99 | MNCN | 39902 | Bolivia, Potosí, Isla, Salar de Uyuni, junto a cerro Pabellón. Daniel Campos | Liolaemus | pachecoi |
| 100 | MNCN | 39903 | Bolivia, Potosí, Isla, Salar de Uyuni, junto a cerro Pabellón. Daniel Campos | Liolaemus | pachecoi |
| 101 | MNCN | 39906 | Bolivia, Potosí, Cerro Pabellón. Salar de Uyuni. Daniel Campos | Liolaemus | pachecoi |
| 102 | MNCN | 39907 | Bolivia, Potosí, Cerro Pabellón. Salar de Uyuni. Daniel Campos | Liolaemus | pachecoi |
| 103 | MNCN | 39909 | Bolivia, Potosí, Isla, Salar de Uyuni, entre Cerro Pabellón y Pescado. Daniel Campos | Liolaemus | pachecoi |
| 104 | MNCN | 39910 | Bolivia, Potosí, Isla, Salar de Uyuni, entre Cerro Pabellón y Pescado. Daniel Campos | Liolaemus | pachecoi |
| 105 | MNCN | 39915 | Bolivia, Potosí, Entre Yonza y San Pedro Quemes. Daniel Campos | Liolaemus | pachecoi |
| 106 | MNCN | 39916 | Bolivia, Potosí, Entre Yonza y San Pedro Quemes. Daniel Campos | Liolaemus | pachecoi |
| 107 | MNCN | 39894 | Bolivia, Potosí, Isla Pescado. Salar de Uyuni. Daniel Campos | Liolaemus | islugensis |
| 108 | MNCN | 39895 | Bolivia, Potosí, Isla Pescado. Salar de Uyuni. Daniel Campos | Liolaemus | islugensis |
| 109 | MNCN | 39912 | Bolivia, Potosí, Cerro Isla Chica, Salar de Uyuni. Daniel Campos | Liolaemus | islugensis |
| 110 | MNCN | 39913 | Bolivia, Potosí, Cerro Isla Chica, Salar de Uyuni. Daniel Campos | Liolaemus | islugensis |
| 111 | MNCN | 48666 | Bolivia, Potosí, Laguna Colorada | Liolaemus | islugensis |
| 112 | MNCN | 48667 | Bolivia, Potosí, Laguna Colorada | Liolaemus | islugensis |
| 113 | MNCN | 48674 | Bolivia, Potosí, Carretera entre San Pablo de Lipez y Tupiza | Liolaemus | islugensis |
| 114 | MNCN | 48506 | Bolivia, La Paz, Cerro Iutane. Lago Titicaca | Liolaemus | signifer |
| 115 | MNCN | 48507 | Bolivia, La Paz, Cerro Iutane. Lago Titicaca | Liolaemus | signifer |
| 116 | MNCN | 48602 | Bolivia, La Paz, Camino a montañas en Millipaya. Sorata | Liolaemus | signifer |
| 117 | MNCN | 5588 | Bolivia, La Paz, Entre Peñas y Amaguaya. Los Andes | Liolaemus | forsteri |
| 118 | MNCN | 5589 | Bolivia, La Paz, Entre Peñas y Amaguaya. Los Andes | Liolaemus | forsteri |
| 119 | MNCN | 5598 | Bolivia, La Paz, Entre Achacachi y Sorata. Omasuyos | Liolaemus | forsteri |
| 120 | MNCN | 34747 | Bolivia, La Paz, Carretera de Zongo, abajo de la represa de Zongo. Provincia Murillo | Liolaemus | forsteri |
| 121 | MNCN | 34748 | Bolivia, La Paz, Carretera de Zongo, abajo de la represa de Zongo. Provincia Murillo | Liolaemus | forsteri |
| 122 | MNCN | 48584 | Bolivia, La Paz, Carretera entre Huarisata y Sorata. Omasuyos. | Liolaemus | forsteri |
| 123 | MNCN | 48585 | Bolivia, La Paz, Carretera entre Huarisata y Sorata. Omasuyos. | Liolaemus | forsteri |
| 124 | MNCN | 48603 | Bolivia, La Paz, Camino a montañas en Millipaya. Sorata | Liolaemus | forsteri |
| 125 | SSUC | 151 | Chile, Atacama, Salar de Pedernales | Liolaemus | rosenmanni |
| 126 | SSUC | 394 | Chile, Atacama, Salar de Pedernales | Liolaemus | rosenmanni |
| 127 | SSUC | 162 | Chile, Atacama, El Cerrito, Salar de Pedernales | Liolaemus | patriciaiturrae |
| 128 | SSUC | 362 | Chile, Antofagasta, Salar de Ascotan | Liolaemus | hajeki |
| 129 | SSUC | 388 | Chile, Antofagasta, Camino a San Pedro | Liolaemus | foxi |
| 130 | SSUC | 569 | Chile, Arica y Parinacoto, Chivatambo | Liolaemus | pleopholis |
| 131 | SSUC | 622 | Chile, Tarapaca, Cerro Isla | Liolaemus | stolzmanni |
| 132 | JT | 98 | Chile, Arica y Parinacoto,, Quebrada de Umirpa | Liolaemus | aymararum |
| 133 | JT | 326 | Chile, Tarapaca, , Camino al Salar de Huasco | Liolaemus | jamesi |
| 134 | JT | 285 | Chile, Atacama, Caseron | Liolaemus | manueli |
| 135 | JT | 328 | Chile, Antofagasta, Cerca del Volcan San Pedro | Liolaemus | islugensis |
| 136 | JT | 327 | Chile, Antofagasta, Cerca del Volcan San Pedro | Liolaemus | islugensis |
| 137 | SSUC | 337 | Chile, Antofagasta, Camino a San Pedro | Liolaemus | cf.schmidti |
| 138 | SSUC | 135 | Chile, Antofagasta, Salar de Aguas Calientes | Liolaemus | cf.schmidti |
| 139 | BYU | 50503 | Peru, Ica, Nazca, Marcona | Ctenoblepharys | adspersa |
| 140 | BYU | 50502 | Peru, Ica, Nazca, Marcona | Ctenoblepharys | adspersa |
| 141 | BYU | 50507 | Peru, Ica, Nazca, Marcona | Liolaemus | "Nazca" |
| 142 | BYU | 50508 | Peru, Ica, Nazca, Marcona | Liolaemus | "Nazca" |
| 143 | BYU | 51569 | Peru, Moquegua, Mariscal Nieto, Jaguay Chico, Desvio carretera a cerro los Calatos | Liolaemus | "Moquegua" |
| 144 | BYU | 51566 | Peru, Moquegua, Mariscal Nieto, Debajo de Otora | Liolaemus | "Moquegua" |
| 145 | BYU | 51568 | Peru, Moquegua, Mariscal Nieto, Jaguay Chico, Desvio carretera a cerro los Calatos | Liolaemus | "Moquegua" |
| 146 | MUSM | 31547 | Peru, Moquegua, Mariscal Nieto, Jaguay Chico, Desvio carretera a cerro los Calatos | Liolaemus | "Moquegua" |
| 147 | MUSM | 31545 | Peru, Tacna, Tacna, Blanquito | Liolaemus | poconchilensis |
| 148 | MUSM | 31543 | Peru, Tacna, Tacna | Liolaemus | poconchilensis |
| 149 | MUSM | 31544 | Peru, Tacna, Uchusuma | Liolaemus | poconchilensis |
| 150 | MUSM | 31490 | Peru, Arequipa, Mollendo, Lomas de Mejía | Liolaemus | insolitus |
| 151 | BYU | 50462 | Peru, Arequipa, Mollendo, Lomas de Mejía | Liolaemus | insolitus |
| 152 | MUSM | 31510 | Peru, Cusco, Paucartambo, Abra Huancarani | Liolaemus | ortizi |
| 153 | MUSM | 31513 | Peru, Cusco, Calca, Chaupimayo | Liolaemus | ortizi |
| 154 | BYU | 50469 | Peru, Cusco, Quipicanchi, Hualla Hualla | Liolaemus | thomasi |
| 155 | BYU | 50466 | Peru, Cusco, Quipicanchi, Hualla Hualla | Liolaemus | thomasi |
| 156 | MUSM | 31504 | Peru, Junín, Junín | Liolaemus | robustus |
| 157 | MUSM | 31508 | Peru, Lima, yauyos | Liolaemus | robustus |
| 158 | BYU | 50438 | Peru, Huancavelica, Huancavelica, Tinyacta | Liolaemus | "MinasMartha" |
| 159 | MUSM | 31446 | Peru, Huancavelica, Huaytara, Santa Inés | Liolaemus | polystictus |
| 160 | MUSM | 31452 | Peru, Huancavelica, Huaytara, Pilpichaca | Liolaemus | polystictus |
| 161 | MUSM | 31455 | Peru, Huancavelica, Castrovirreyna, Sinto | Liolaemus | "Castrovirreyna" |
| 162 | MUSM | 31481 | Peru, Ayacucho, Huamanga, Abra Apacheta | Liolaemus | "AbraApacheta" |
| 163 | MUSM | 31371 | Peru, Ayacucho, Huamanga, Comunidad deToccto | Liolaemus | "AbraToccto" |
| 164 | MUSM | 31374 | Peru, Ayacucho, Huamanga, Comunidad deToccto | Liolaemus | "AbraToccto" |
| 165 | BYU | 50426 | Peru, Ayacucho, Huamanga, Abra Toccto | Liolaemus | "AbraToccto" |
| 166 | MUSM | 31461 | Peru, Ayacucho, Huamanga, Abra Toccto | Liolaemus | "AbraToccto" |
| 167 | MUSM | 31373 | Peru, Ayacucho, Huamanga, Comunidad deToccto | Liolaemus | "AbraToccto" |
| 168 | BYU | 50151 | Peru, Ayacucho, Lucanas, Abra Apacheta, Km 45 desde Puquio | Liolaemus | melanogaster |
| 169 | BYU | 50152 | Peru, Ayacucho, Lucanas, Abra Apacheta, Km 45 desde Puquio | Liolaemus | melanogaster |
| 170 | BYU | 50463 | Peru, Ayacucho, Lucanas, Lucanas | Liolaemus | williamsi |
| 171 | MUSM | 31485 | Peru, Ayacucho, Lucanas, Lucanas | Liolaemus | williamsi |
| 172 | BYU | 50489 | Peru, Arequipa, Arequipa, Sumbay | Liolaemus | annectens |
| 173 | BYU | 50486 | Peru, Arequipa, Caylloma | Liolaemus | annectens |
| 174 | BYU | 50494 | Peru, Arequipa, Arequipa, Pocsi | Liolaemus | etheridgei |
| 175 | BYU | 50495 | Peru, Arequipa, Arequipa, Pocsi | Liolaemus | etheridgei |
| 176 | MUSM | 31443 | Peru, Puno, Lago Titicaca, Isla Amantani | Liolaemus | signifer |
| 177 | MUSM | 31434 | Peru, Puno, Lago Titicaca, Isla Amantani | Liolaemus | signifer |
| 178 | MUSM | 27688 | Peru, Apurimac, Cotabambas, Huasijasa | Liolaemus | "Apurimac" |
| 179 | MUSM | 27694 | Peru, Apurimac, Cotabambas, progreso | Liolaemus | "Apurimac" |
| 180 | MUSM | 26393 | Peru, Ayacucho, Parinacochas, Chumpi | Liolaemus | "Parinacochas" |
| 181 | MUSM | 26387 | Peru, Ayacucho, Parinacochas, Chumpi | Liolaemus | "Parinacochas" |

**References**

**Aguilar C., Wood Jr. P.L., Belk M.C., Duff M.H., Sites Jr.,J.W. 2017.** Different roads lead to Rome: Integrative taxonomic approaches lead to the discovery of two new lizard lineages in the *Liolaemus* montanus group (Squamata: Liolaemidae). *Biological Journal of the Linnean Society* **120:** 448–467.

**Corl A., Davis A.R, Kuchta S.R, Comendant T, Sinervo B. 2010.** Alternative mating strategies and the evolution of sexual size dimorphism in the side-blotched lizard, *Uta stansburiana*: a population-level comparative analysis. *Evolution* **64:** 79**–**96.

**Hillis D.M., Bull J.J. 1993.** An empirical test of bootstrapping as a method for assessing confidence in phylogenetic analysis. *Systematic Biology* **42:** 182–192.

**Lobo F., Espinoza R.E., Quinteros S. 2010.** A critical review and systematic discussion of recent classification proposals for liolaemid lizards. *Zootaxa* **2549:** 1–30.

**Portik D.M, Wood P.L Jr, Grismer J.L, Stanley E.L, Jackman T.R. 2012.** Identification of 104 rapidly-evolving nuclear protein-coding markers for amplification across scaled reptiles using genomic resources. *Conservation Genetics Resources* **4:** 1**–**10.

**Townsend T.M., Alegre R.E., Kelley S.T., Wiens J.J., Reeder T.W. 2008.** Rapid developmental of multiple nuclear loci or phylogenetic analysis using genomic resources: an example from squamate reptiles. *Molecular Phylogenetics and Evolution* **47:** 129**–**142.

**Wiens J.J., Reeder T.W., Montes de Oca A.N.1999.** Molecular phylogenetics and evolution in sexual dichromatism among population of the Yarrow**’**s spiny lizard (*Sceloporus jarrovii*). *Evolution* **53:**1884**–**1897.

**Wiens J.J., Hutter C.R., Mulcahy D.G., Noonan B.P., Townsend T.M., Sites J.W., Reeder T.W. 2012.** Resolving the phylogeny of lizards and snakes (Squamata) with extensive sampling of genes and species. *Biology Letters* rsbl20120703.
