# Supplementary material for: The shadow of the past: Convergence of young and old South American desert lizards as measured by head shape traits
Source: Ecol Evol. 2018 Nov 26;8(23):11399–409. doi: 10.1002/ece3.4548 (PMC6303702; doi:10.1002/ece3.4548)
Supplement: Supplementary file 3 [file ECE3-8-11399-s003.docx]

**Supplementary Material 2**

GenBank accession numbers of sequences used in this study. Blank spaces are unavailable sequences

| **Terminal_Museum Number** | **GenBank accesion number by marker** | | | | | | |
| --- | --- | --- | --- | --- | --- | --- | --- |
|  | **12S** | **CYTB** | **CMOS** | **EXPH5** | **KIF24** | **MXRA5** | **PRLR** |
| Liolaemus_Nazca_BYU_50507 | KX826769 | KX826674 | MH981349 | KX826582 | KX826714 | KX826609 | MH981549 |
| Liolaemus_poconchilensis_MUSM_31544 | KX826735 | KX826636 | MH981360 | KX826567 | KX826698 | KX826595 | MH981568 |
| Liolaemus_insolitus_MUSM_31490 | KX826727 | KX826627 | MH981351 | KX826562 | MH981466 | KX826591 | MH981554 |
| Liolaemus_Moquegua_BYU_51568 | MH888057 | MH981365 | MH981361 | MH981436 | MH981459 | MH981512 | MH981582 |
| Liolaemus_ortizi_MUSM_31513 | KX826733 | KX826633 | MH981333 | KX826565 | KX826696 | KX826593 | MH981540 |
| Liolaemus_thomasi_BYU_50469 | KX826775 | KX826680 | MH981319 | KX826585 | KX826715 | KX826612 | MH981541 |
| Liolaemus_robustus_MUSM_31504 | KX826743 | KX826646 | MH981335 | KX826574 | MH981469 | KX826600 | MH981560 |
| Liolaemus_MinaMartha_BYU_50438 | KX826741 | KX826644 | MH981336 | KX826573 | KX826702 | KX826599 | MH981561 |
| Liolaemus_polystictus_MUSM_31446 | KX826739 | KX826641 | MH981357 | KX826571 | KX826701 | KX826598 | MH981587 |
| Liolaemus_AbraApacheta_MUSM_31481 | KX826756 | KX826660 | MH981310 | KX826577 | KX826708 | KX826604 | MH981592 |
| Liolaemus _Castrovirreyna_MUSM_31455 |  | KX826640 | MH981337 | KX826570 | KX826700 | KX826597 | MH981590 |
| Liolaemus_AbraToccto_MUSM_31374 | KX826762 | KX826667 | MH981339 | KX826580 | KX826712 | KX826607 | MH981584 |
| Liolaemus_melanogaster_BYU_50151 | KX826728 | KX826628 |  | KX826563 | KX826694 | KX826592 | MH981583 |
| Liolaemus_williamsi_BYU_50463 | KX826778 | KX826684 | MH981309 | KX826586 |  |  | MH981585 |
| Liolaemus_annectens_BYU_50489 | KX826717 | KX826616 | MH981334 | KX826559 | KX826689 | MH981529 | MH981553 |
| Liolaemus_etheridgei_BYU_50494 | KX826721 | KX826620 | MH981354 | KX826560 | KX826691 | KX826589 | MH981557 |
| Liolaemus_Apurimac_MUSM_27694 | MH888042 | MH981371 | MH981329 | MH981417 | MH981473 | MH981506 | MH981562 |
| Liolaemus_Parinacochas_MUSM_26393 | MH888062 | MH981370 | MH981341 | MH981418 | MH981460 | MH981502 | MH981563 |
| Liolaemus_signifer_MUSM_31434 | KX826750 | KX826654 | MH981322 | KX826575 | KX826706 | KX826603 | MH981550 |
| Liolaemus_andinus_LJAMMCNP_14394 | MH888041 | MH981378 | MH981352 | MH981431 | MH981492 | MH981521 | MH981556 |
| Liolaemus_dorbigny_LJAMMCNP_5018 | MH888047 | MH981392 | MH981315 | MH981438 | MH981478 | MH981517 | MH981552 |
| Liolaemus_porosus_LJAMMCNP_15690 |  | MH981395 | MH981346 | MH981444 | MH981456 | MH981514 | MH981576 |
| Liolaemus_poecilochromus_LJAMMCNP_14735 | MH888065 | MH981399 | MH981324 | MH981427 | MH981474 | MH981500 | MH981547 |
| Liolaemus_gracielae_LJAMMCNP_12555 |  | MH981374 | MH981317 | MH981413 | MH981488 | MH981520 |  |
| Liolaemus_multicolor_LJAMMCNP_12008 | MH888058 | MH981381 | MH981318 | MH981429 | MH981465 | MH981522 | MH981548 |
| Liolaemus_ruibali_LJAMMCNP_13978 | MH888067 | MH981387 | MH981347 | MH981411 | MH981489 | MH981518 | MH981543 |
| Liolaemus_chlorostictus_LJAMMCNP_16055 | MH888046 | MH981369 | MH981338 | MH981415 |  | MH981531 | MH981589 |
| Liolaemus_huacahuasicus_LJAMMCNP_12829 | MH888053 | MH981379 | MH981343 | MH981433 | MH981485 | MH981516 | MH981564 |
| Liolaemus_nigriceps_LJAMMCNP_15761 | MH888059 | MH981376 | MH981348 | MH981422 | MH981491 | MH981497 | MH981569 |
| Liolaemus_inti_LJAMMCNP_15664 | MH888054 | MH981394 | MH981359 | MH981409 | MH981484 | MH981507 | MH981578 |
| Liolaemus_fittkaui_ MNCN _59257 | MH888049 | MH981398 | MH981344 | MH981435 | MH981457 | MH981499 | MH981542 |
| Liolaemus_cazianiae_LJAMMCNP_15665 | MH888045 | MH981396 | MH981314 | MH981443 | MH981476 |  | MH981574 |
| Liolaemus_orientalis_8406 | MH888060 | MH981382 | MH981325 | MH981416 | MH981470 | MH981530 | MH981588 |
| Liolaemus_vallecurensis_LJAMMCNP_2709 | MH888075 | MH981373 | MH981316 | MH981408 | MH981490 | MH981495 | MH981593 |
| Liolaemus_famatinae_LJAMMCNP_2034 | MH888048 | MH981377 | MH981332 | MH981419 | MH981455 | MH981505 | MH981579 |
| Liolaemus_halonastes_LJAMMCNP_15792 | MH888052 | MH981400 | MH981326 | MH981430 | MH981475 | MH981501 | MH981577 |
| Liolaemus_scrocchii_LJAMMCNP_15803 | MH888068 | MH981393 | MH981313 | MH981410 | MH981482 | MH981510 | MH981551 |
| Liolaemus_vulcanus_LJAMMCNP_14736 | MH888076 | MH981391 | MH981311 | MH981407 | MH981477 | MH981515 | MH981573 |
| Liolaemus_pachecoi_MNCN_39916 | MH888061 | MH981402 | MH981331 | MH981421 | MH981458 | MH981511 | MH981581 |
| Liolaemus_islugensis_MNCN_48674 | MH888055 | MH981386 | MH981353 | MH981437 | MH981471 | MH981496 | MH981567 |
| Liolaemus_forsteri_MNCN_48603 | MH888050 | MH981390 | MH981350 | MH981414 | MH981467 | MH981527 | MH981572 |
| Liolaemus_rosenmanni_SSUC_151 | MH888066 | MH981372 | MH981340 | MH981426 | MH981486 | MH981519 | MH981586 |
| Liolaemus_patriciaiturrae_SSUC_162 | MH888063 | MH981375 |  | MH981412 | MH981487 |  | MH981580 |
| Liolaemus_hajeki_SSUC_163 |  | MH981404 |  |  |  |  |  |
| Liolaemus_foxi_SSUC_388 | MH888051 |  |  |  |  |  |  |
| Liolaemus_pleopholis_SSUC_569 | MH888064 | MH981385 |  |  |  |  |  |
| Liolaemus_stolzmanni_SSUC_622 |  | MH981397 | MH981345 | MH981442 | MH981479 | MH981509 |  |
| Liolaemus_cf_schmidti_SSUC_135 |  | MH981384 | MH981358 | MH981441 | MH981468 | MH981528 | MH981558 |
| Liolaemus_aymararum_JT_98 | MH888044 | MH981401 | MH981327 | MH981423 | MH981481 | MH981503 | MH981566 |
| Liolaemus_jamesi_JT_326 | MH888056 | MH981403 | MH981342 | MH981420 | MH981480 | MH981513 | MH981565 |
| Liolaemus_audituvelatus_JT_285 | MH888043 | MH981367 | MH981328 | MH981434 | MH981493 | MH981504 | MH981591 |
| Liolaemus_sp1_LJAMMCNP_16033 | MH888069 | MH981380 | MH981321 | MH981439 | MH981464 | MH981525 | MH981570 |
| Liolaemus_sp2_MNCN_48613_Torohuaico | MH888071 | MH981389 |  | MH981424 | MH981462 | MH981526 | MH981555 |
| Liolaemus_sp2_8396_Sama | MH888070 | MH981366 | MH981323 | MH981425 | MH981463 | MH981498 | MH981571 |
| Liolaemus_sp3_MNCN_48572 | MH888072 | MH981368 | MH981330 | MH981432 | MH981472 | MH981523 | MH981546 |
| Liolaemus_sp4_LJAMMCNP_12471 | MH888073 | MH981383 | MH981320 | MH981428 | MH981461 | MH981524 | MH981559 |
| Liolaemus_sp5_LJAMMCNP_14730 | MH888074 | MH981388 | MH981312 | MH981440 | MH981483 | MH981508 | MH981575 |
| Liolaemus_ornatus_LJAMMCNP_12021 | KF969090 | KF968895 | KF968713 | KF968330 | KF968149 | KF968001 | KF967640 |
| Liolaemus_lentus_LJAMMCNP_12851 | KF968871 | KF969060 | KF968686 |  | KF968120 |  | KF967610 |
| Liolaemus_baguali_LJAMMCNP_9394 | KF969008 | KP175414 | KF968635 | KF968264 | KF968072 | KF967949 | JF272898 |
| Liolaemus_canqueli_LJAMMCNP_4665 | KF969017 |  | KF968645 | KF968271 | KF968080 | KF967955 | KF967568 |
| Liolaemus_puelche_LJAMMCNP 5759 | KF969096 |  | KF968719 |  | KF968155 |  | KF967647 |
| Liolaemus_rothi_LJAMMCNP_12941 | KF969198 | KF968991 | KF968815 | KF968422 | KF968252 | KF968058 | KF967748 |
| Liolaemus_walkeri_BYU_50340 | KF923674 | KF923646 |  |  |  |  |  |
| Liolaemus_walkeri_BYU_50342 |  |  | MH981362 | MH981447 | MH981453 | MH981532 | MH981544 |
| Liolaemus_tacnae_MUSM_29607 | KF923670 | KF923642 |  |  |  |  |  |
| Liolaemus_tacnae_MUSM_31406 |  |  | MH981363 | MH981448 | MH981454 | MH981533 | MH981545 |
| Liolaemus_alticolor_MUSM_29109 | KF923687 | KF923659 | MH981356 | MH981446 | MH981452 | MH981535 | MH981539 |
| Liolaemus_incaicus_MUSM_31398 | KF923685 | KF923657 | MH981355 | MH981445 | MH981451 | MH981534 | MH981538 |
| Phymaturus_sitesi_LJAMMCNP_10367 | JX969100 | JX969049 | X969553 |  |  |  |  |
| Phymaturus_sitesi_LJAMMCNP_12190 |  |  |  | MH981406 | MH981450 |  | MH981537 |
| Ctenoblepharys_adspersa_BYU_50503 | MH888040 |  |  | MH981405 | MH981449 | MH981494 | MH981536 |
| Ctenoblepharys_adspersa_BYU_50502 |  | MH981364 |  |  |  |  |  |
| Phrynosoma_platyrhinos | DQ385401 | AY141093 |  | KJ124045 |  |  | GQ896081 |
| Anolis_carolinensis | AF339050 | EU747728 |  |  |  |  | JN880807 |
| Chamaeleo_calyptratus | NC_012420 | EF222192 | HF570667 |  |  |  | JN880819 |
