# Supplementary material for: The shadow of the past: Convergence of young and old South American desert lizards as measured by head shape traits
Source: Ecol Evol. 2018 Nov 26;8(23):11399–409. doi: 10.1002/ece3.4548 (PMC6303702; doi:10.1002/ece3.4548)
Supplement: Supplementary file 4 [file ECE3-8-11399-s004.docx]

**Supplementary Material 3.**

Landmarks used in shape analyses.


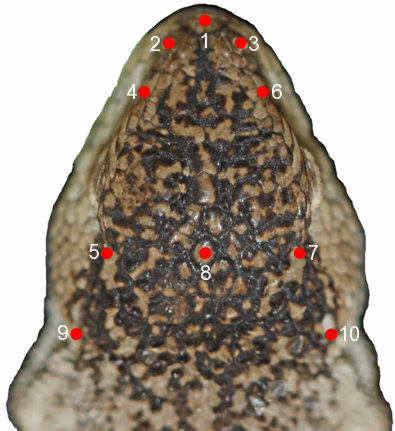


Principal component eigenvalues and variance

| PC | Eigenvalues | % Variance | Cumulative % |
| --- | --- | --- | --- |
| 1 | 0,00221088 | 36,214 | 36,214 |
| 2 | 0,00144054 | 23,596 | 59,810 |
| 3 | 0,00091467 | 14,982 | 74,792 |

List of species used in convergence analyses, number of specimens and principal component scores (PC1-PC3)

| Genus | species/candidate species | Number of specimens | PC1 | PC2 | PC3 |
| --- | --- | --- | --- | --- | --- |
| Ctenoblepharys | adspersa | 6 | -0.118498529 | -0.03850184 | 0.014235249 |
| Liolaemus | "AbraApacheta" | 10 | -0.010405656 | 0.014695376 | -0.022122353 |
| Liolaemus | "AbraToccto" | 24 | -0.001018942 | 0.044575484 | 0.013106844 |
| Liolaemus | andinus | 7 | 0.045138641 | -0.037643047 | 0.009169474 |
| Liolaemus | annectens | 35 | 0.076261095 | 0.006512125 | -0.009314516 |
| Liolaemus | "Apurimac" | 31 | 0.043919402 | 0.011811478 | -0.000587055 |
| Liolaemus | aymararum | 9 | 0.011957266 | -0.007594469 | 0.003908864 |
| Liolaemus | baguali | 2 | -0.048759398 | 0.00968202 | 0.020692967 |
| Liolaemus | canqueli | 2 | -0.047047046 | 0.01774993 | 0.038080155 |
| Liolaemus | "Castrovirreyna" | 1 | -0.045398848 | 0.058890108 | 0.030966225 |
| Liolaemus | cazianae | 2 | -0.006197062 | -0.022772814 | 0.032127805 |
| Liolaemus | chlorostictus | 4 | -0.011787877 | -0.005392577 | 0.032118999 |
| Liolaemus | dorbignyi | 4 | 0.067318922 | -0.02149999 | -0.021508608 |
| Liolaemus | etheridgei | 9 | -0.019026311 | 0.022929169 | 0.027241854 |
| Liolaemus | fittkaui | 2 | -0.010795989 | 0.043698446 | 0.033619883 |
| Liolaemus | forsteri | 9 | -0.002745899 | 0.010355522 | -0.007880295 |
| Liolaemus | foxi | 9 | 0.02308876 | -0.037398439 | 0.018747093 |
| Liolaemus | hajeki | 2 | 0.026645729 | -0.036229199 | 0.011063456 |
| Liolaemus | halonastes | 2 | 0.031088597 | -0.041697828 | 0.02590528 |
| Liolaemus | huacahuasicus | 7 | 0.036999595 | -0.025102575 | 0.01303512 |
| Liolaemus | insolitus | 9 | -0.037047605 | -0.016501652 | 0.039994531 |
| Liolaemus | islugensis | 16 | -0.02961424 | -0.032416428 | -0.012747899 |
| Liolaemus | jamesi | 2 | 0.019468074 | -0.026428593 | 0.035411899 |
| Liolaemus | lentus | 1 | -0.060631264 | -0.045474014 | -0.054593444 |
| Liolaemus | manueli | 11 | -0.044692379 | -0.09787906 | 0.005399889 |
| Liolaemus | melanogaster | 9 | -0.011386744 | 0.022570401 | -0.014674836 |
| Liolaemus | "MinasMartha" | 9 | 0.076101643 | -0.019155577 | 0.018926716 |
| Liolaemus | "Moquegua" | 9 | -0.087118769 | -0.033926474 | -0.000549977 |
| Liolaemus | multicolor | 10 | 0.057300982 | -0.035887613 | 0.028606837 |
| Liolaemus | "Nazca" | 10 | -0.009977604 | 0.01777858 | -0.025092725 |
| Liolaemus | nigriceps | 11 | 0.010255871 | -0.0250449 | 0.013725345 |
| Liolaemus | orientalis | 3 | -0.02305316 | 0.018417173 | -0.048571532 |
| Liolaemus | ornatus | 4 | -0.03487823 | 0.020004668 | 0.024021858 |
| Liolaemus | ortizi | 8 | -0.025247718 | 0.047283192 | -0.018765089 |
| Liolaemus | pachecoi | 12 | -0.031877468 | -0.01585071 | -0.014682259 |
| Liolaemus | sp2 | 4 | -0.049771805 | -0.017881956 | -0.0374502 |
| Liolaemus | "Parinacochas" | 12 | -0.020572913 | 0.03851591 | 0.004200931 |
| Liolaemus | patriciaiturrae | 5 | 0.043705959 | -0.031318102 | 0.00850811 |
| Liolaemus | pleopholis | 1 | 0.057153128 | -0.046904892 | 0.010547219 |
| Liolaemus | poconchilensis | 9 | -0.062110036 | -0.057370453 | 0.0229737 |
| Liolaemus | poecilochromus | 11 | 0.016374746 | -0.029046198 | 0.012345653 |
| Liolaemus | polystictus | 37 | -0.006444991 | 0.021460887 | -0.025537113 |
| Liolaemus | porosus | 6 | 0.040769325 | -0.041648129 | -0.015009088 |
| Liolaemus | robustus | 20 | -0.005299163 | 0.038000774 | 0.010503515 |
| Liolaemus | rosenmanni | 1 | 0.078087012 | -0.076215937 | -0.011458812 |
| Liolaemus | rothi | 3 | -0.050982552 | 0.024004725 | 0.018049275 |
| Liolaemus | scrocchii | 1 | -0.045515478 | -0.02524197 | -0.006753778 |
| Liolaemus | signifer | 13 | 0.005104515 | 0.018093682 | 0.003242814 |
| Liolaemus | sp3 | 10 | -0.023157314 | 0.007446843 | -0.01491614 |
| Liolaemus | sp5 | 1 | 0.040345311 | -0.026476227 | 0.009265802 |
| Liolaemus | stolzmanni | 6 | -0.055413631 | -0.060889132 | 0.057081123 |
| Liolaemus | thomasi | 4 | 0.019175723 | 0.051260768 | -0.006901081 |
| Liolaemus | vallecurensis | 2 | 0.034899228 | -0.015838084 | 0.025026544 |
| Liolaemus | vulcanus | 2 | 0.050185368 | -0.021058112 | -0.027311649 |
| Liolaemus | walkeri | 5 | -0.022772975 | 0.031621895 | 0.011088638 |
| Liolaemus | williamsi | 12 | -0.027112127 | 0.031254526 | -0.018411877 |
| Phymaturus | patagonicus | 2 | -0.06658327 | -0.061139036 | -0.09360956 |

Test of symmetry and Procrustes ANOVA

We found significant variation between individuals (shape differences) and variation between head sides.. SS (sum of squares), MS (Mean squares), df (degrees of freedom), F statistic (F), and parametric *P* values are shown. In addition, a MANOVA test shown significant variation between head sides. Pillai trace (Pillai tr) and parametric *P* values are also shown.

Centroid size:

Effect SS MS df F *P* (param.)

Individual 1057114308,35 2263628,06 467

Shape, Procrustes ANOVA:

Effect SS MS df F *P* (param.)

Individual 2,56527367 0,0006866364 3736 10,95 <.0001

Side 0,00512088 0,0006401103 8 10,21 <.0001

Ind * Side 0,23422572 0,0000626943 3736

Shape, MANOVA tests of effects:

Symmetric component of shape variation:

Effect Pillai tr. *P* (param.)

Note: the test for 'Individual' used the symmetric component of the residual as the 'error' effect.

Asymmetry component of shape variation:

Effect Pillai tr. *P* (param.)

Side 0,10 <.0001

Test of between sex differences in head shape: Discriminant Function Analysis

Comparison: Females -- Males

Difference between means:

Procrustes distance: 0,01420524

Mahalanobis distance: 0,5020

T-square: 28,3530, *P*-value (parametric): 0,0415

*P*-values for permutation tests (1000 permutation runs):

Procrustes distance: 0,0050

T-square: 0,0510

(Note: The permutation test using the T-square statistic is equivalent to a test using Mahalanobis distance.)
